# Molecular Merged Hypergraph Neural Network for Explainable Solvation Gibbs Free Energy Prediction

**DOI:** 10.34133/research.0740

**Published:** 2025-08-15

**Authors:** Wenjie Du, Shuai Zhang, Zhaohui Cai, Xuqiang Li, Zhiyuan Liu, Junfeng Fang, Jianmin Wang, Xiang Wang, Yang Wang

**Affiliations:** ^1^ Key Laboratory of Precision and Intelligent Chemistry, University of Science and Technology of China, Hefei, Anhui 230026, China.; ^2^ Suzhou Institute for Advanced Research, University of Science and Technology of China, Suzhou, Jiangsu 215123, China.; ^3^ Suzhou Laboratory, Suzhou 215000, China.; ^4^ School of Advanced Technology, Xi’an Jiaotong–Liverpool University, Suzhou 215123, China.; ^5^ School of Computing, National University of Singapore, Singapore 117417, Singapore.; ^6^ Department of Integrative Biotechnology, Yonsei University, Incheon 21983, Republic of Korea.; ^7^ Anhui Provincial Key Laboratory of High Performance Computing, Hefei, China.

## Abstract

Solvation free energies play a fundamental role in various fields of chemistry and biology. Accurately determining the solvation Gibbs free energy ($\Delta G_{\text{solv}}$) of a molecule in a given solvent requires a deep understanding of the intrinsic relationships between solute and solvent molecules. While deep learning methods have been developed for $\Delta G_{\text{solv}}$ prediction, few explicitly model intermolecular interactions between solute and solvent molecules. The molecular modeling graph neural network more closely aligns with real-world chemical processes by explicitly capturing atomic-level interactions, such as hydrogen bonding. It achieves this by initially establishing indiscriminate connections between intermolecular atoms, which are subsequently refined using an attention-based aggregation mechanism tailored to specific solute–solvent pairs. However, its sharply increasing computational complexity limits its scalability and broader applicability. Here, we introduce an improved framework, molecular merged hypergraph neural network (MMHNN), which leverages a predefined subgraph set and replaces subgraphs with supernodes to construct a hypergraph representation. This design effectively mitigates model complexity while preserving key molecular interactions. Furthermore, to handle noninteractive or repulsive atomic interactions, MMHNN incorporates an interpretation mechanism for nodes and edges within the merged graph, leveraging the graph information bottleneck theory to enhance model explainability. Extensive experimental validation demonstrates the efficiency of MMHNN and its improved interpretability in capturing solute–solvent interactions.

## Introduction

The interaction properties of solute–solvent in a specific solvent plays a crucial role in physical chemistry, such as chemical reactions [1,2] and electrochemistry [3], among others. A profound understanding of these interactions is essential not only for rationalizing experimental results, but also for guiding the way toward controlling and designing reactions and properties [4,5]. The solvation Gibbs free energy ($\Delta G_{\text{solv}}$) is a crucial physicochemical property that determines the behavior of a molecule in solution [6] and is closely linked to the partition coefficient of the solute between the gas and solvent phase [4,7]. In realistic applications, obtaining an accurate $\Delta G_{\text{solv}}$ result from a large number of solute–solvent combinations can be challenging and expensive using advanced-level methods such as first-principles calculations. Furthermore, changes in solvent and/or solute environment require to be recalculated and reevaluated [1,3,8–10]. Deep learning could serve as an effective tool that has emerged to predict $\Delta G_{\text{solv}}$ in different solute–solvent systems, providing results quickly without seriously sacrificing accuracy in many cases even when experimental measurements are difficult to obtain.

**Fig. 1.**
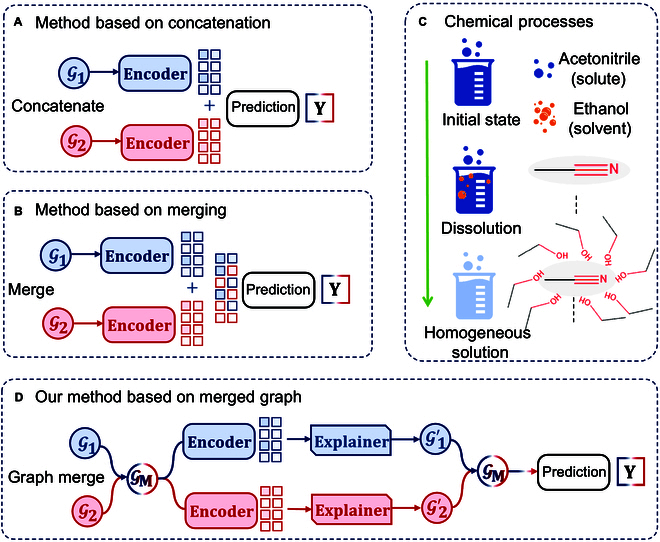
Illustration of different paradigms for $\Delta G_{\text{solv}}$ prediction. (A) Embedding concatenation; (B) embedding merging; (C) a schematic diagram of the dissolution process, where acetonitrile is dissolved in ethanol through intermolecular interactions; and (D) our proposed method.

A wide range of methods have been developed [6,11,12] for $\Delta G_{\text{solv}}$ prediction, with solute–solvent interaction modeling predominantly relying on 2 key approaches. The first, embedding concatenation, utilizes separate graph neural networks (GNNs) to encode solute and solvent molecules independently. The resulting embeddings are then concatenated for downstream prediction, as illustrated in Fig. 1A. While straightforward, this approach primarily captures intramolecular representations at the node and edge levels within individual molecules, potentially neglecting critical intermolecular interactions that govern solvation behavior. In contrast, the embedding merging, depicted in Fig. 1B, seeks to bridge this gap by explicitly modeling solute–solvent interactions. Like the previous method, it employs 2 graph encoders for initial molecular representation. However, instead of direct concatenation, the embeddings undergo structured integration via advanced interaction mechanisms, such as transformer-based architectures or interactive pruning algorithms, ensuring a more comprehensive reflection of complex intermolecular coupling before prediction. This enhanced representation framework better captures the nuances of solvation free energy, offering a more faithful approximation of molecular interactions in solution.

While the embedding merging approach represents a step forward, it remains insufficient for accurately capturing the underlying chemical processes governing dissolution, as illustrated in Fig. 1C. Dissolution fundamentally involves the replacement of intramolecular forces within the solute and solvent by intermolecular interactions between them. A representative example is the dissolution of acetonitrile in ethanol, where acetonitrile molecules become progressively surrounded by ethanol, forming a homogeneous solution through hydrogen bonding at the atomic (node) level. This natural process underscores the necessity of explicitly modeling intermolecular node interactions, rather than relying solely on embedding-level representations. A model that directly encodes these fine-grained interactions would more faithfully reflect real chemical behavior, enhancing both scientific rigor and predictive accuracy in solvation free energy estimation [13–15].

In order to address to these limitations, we propose the molecular modeling graph neural network (MMGNN) in International Joint Conference on Artificial Intelligence (IJCAI) 2024 [16], which is a novel framework to simulate the actual chemical processes in solute–solvent interactions. MMGNN substantially improves the prediction accuracy of $\Delta G_{\text{solv}}$ as illustrated in Fig. 1D. Our model has achieved state-of-the-art (SOTA) performance across multiple benchmark datasets, demonstrating superior generalization capability and interpretability [16]. However, we observe 2 critical challenges. First, the indiscriminate construction of intermolecular edges leads to a rapid increase in computational complexity, particularly for large molecules. Second, treating solute and solvent with the same information propagation strategy may mislead the model, making it unable to distinguish between chemically distinct scenarios, such as dissolving ethanol in water versus dissolving water in ethanol, where the roles of solute and solvent are fundamentally different. Addressing these challenges is essential for further improving both the efficiency and robustness of MMGNN in complex chemical environments.

**Fig. 2.**
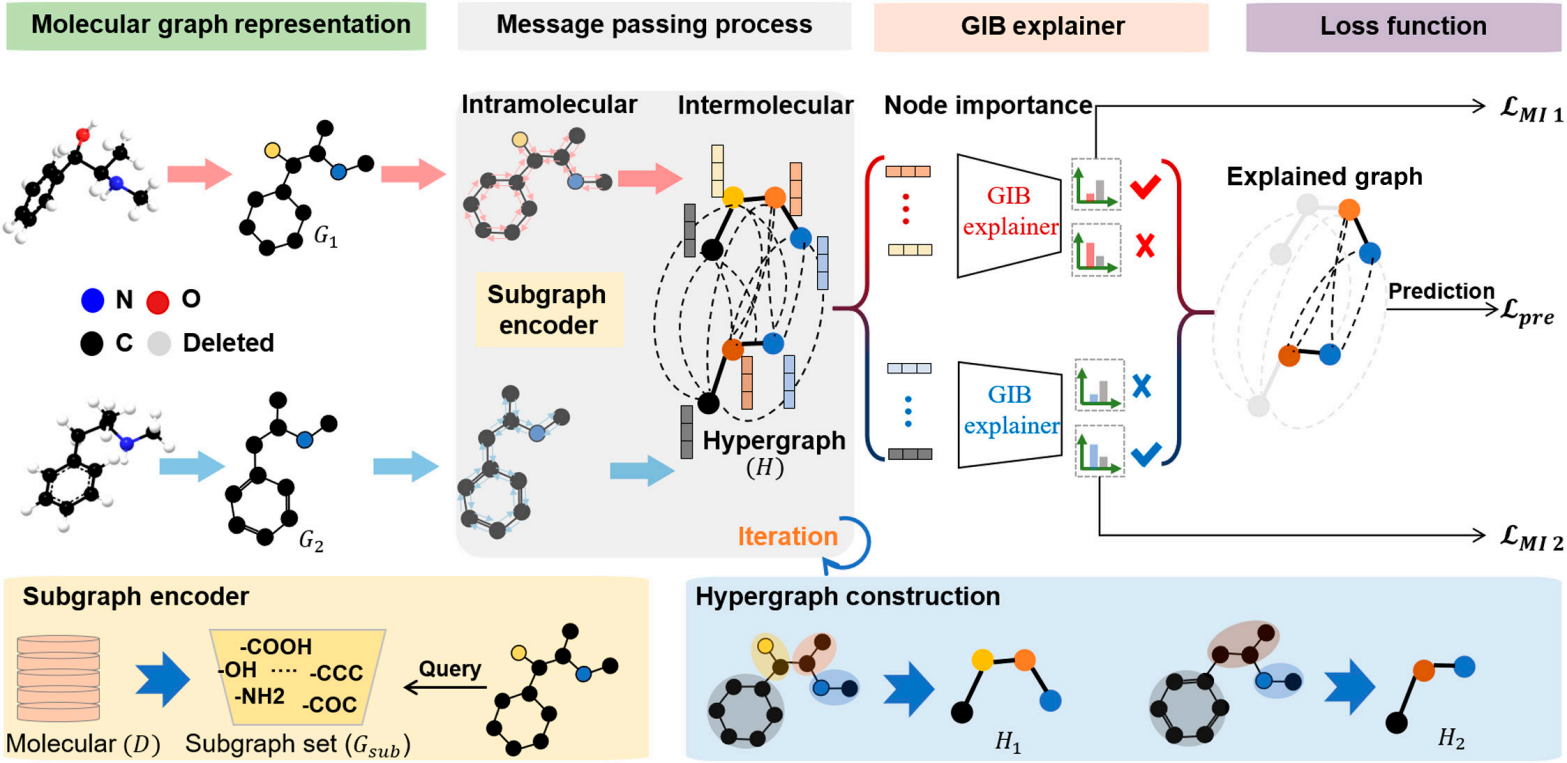
Overview of the enhanced merged hypergraph neural network model. The model begins by converting an input molecular pair into a topological graph. Substructures are mined and treated as hypernodes, forming a hypergraph to facilitate inter- and intramolecular message passing. The initial layer updates solute atomic features using classical GNN methods, followed by a cross-molecular co-attention mechanism based on hypernodes to refine interactions. Finally, a graph information bottleneck (GIB) principle is applied to highlight significant substructures, improving model interpretability and alignment with biochemical realities. Best viewed in color.

Hence, we further propose an optimized evaluation method, molecular merged hypergraph neural network (MMHNN), building upon the foundation of MMGNN. Specifically, as depicted within Fig. 2, MMHNN incorporates the following optimizations:

• Predefined subgraph set: The current practice of assigning weights to relationship edges only considers atomic interactions. However, in practical chemical processes such as solvation or binding, spontaneous molecular interactions occur, where van der Waals forces and hydrogen bonding play significant roles. Certain specific atomic and subgraph combinations, such as -OH, -COOH, N, O, F, H, etc. [17–19], exhibit stronger interactions. To address this, we construct a prior knowledge repository to enhance attention mechanisms, allowing for not only the dynamic adjustment of relationship edge weights but also the consideration of interactions between atoms, atom–subgraph, and subgraph–subgraph relationships.
• Merged hypergraph neural network: Constructing relationship edges in pairs, as previously done, is an effective strategy for predicting properties between solute–solvent molecular pairs. However, this approach significantly increases the graph’s complexity, especially for large, intricate molecules, due to the substantial rise in degrees of freedom, leading to higher computational resource and time consumption. To address this challenge, we propose a merged hypergraph structure, where a super node aggregates molecular frequency subgraph information using a molecular autoencoder. Information transmission between super nodes facilitates intermolecular message passing, effectively mitigating the issue.
• Explainable method based on GIB: In the merged hypergraph, hypernodes encapsulate critical substructure information. Inspired by graph explainability algorithms, we employ an enhanced information bottleneck theory to derive interpretable results on the hypergraph. MMHNN incorporates interpreters for hypernodes in the merged graph, which aims to reduce redundancy and concentrate on pertinent interactions. The explanatory subgraph is then encoded and used to predict Gibbs free energy with improved accuracy.

A preliminary version of this work was published in IJCAI 2024 [16]. This journal manuscript (MMHNN) extends the initial version in several key aspects. Firstly, MMHNN significantly broadens the application scope and enhances the reliability of MMGNN by introducing a hypergraph construction and a prior knowledge-based reweighting block. Secondly, we extend our investigation to include a variety of solvation free energy prediction tasks, utilizing a range of both molecular interaction and intrinsic explanation methods, aiming for a comprehensive validation of fairness and reliability. Thirdly, considering the computational complexity and time consumption of the model, we evaluated the complexity and runtime of different models, providing a comprehensive assessment of their performance.

## Results and Discussion

### Overview of the model

The improved novel merged molecular graph model introduces a hypergraph concept where nodes represent substructures, enabling enhanced molecular interactions as shown in Fig. 2. Specifically, a solute–solvent pair is firstly converted into a machine-readable topological graph, as detailed in the “Molecular graph representation” section. We then apply frequent subgraph mining to extract substructures, construct a subgraph set, and treat these as hypernodes to form a hypergraph (see the “Hypergraph construction” section). Within this hypergraph, we facilitate both inter- and intramolecular message passing processes. In the initial layer of message passing, each atomic feature of the solute molecule is updated independently using classical GNN methods. Subsequently, a cross-molecular co-attention mechanism based on hypernodes is employed to further update the interaction graph nodes in the “Intramolecular message passing process” and “Intermolecular message passing process” sections. Lastly, to enhance interpretability, we incorporate graph information bottleneck (GIB) principle to capture a core subgraph, as presented in the “Core hypernode extraction based on GIB” section, highlighting the most significant atomic interactions. This augmentation aims to improve the model’s generalization capability and better align it with biochemical realities.

A comprehensive overview of the selected atom, bond, and global input features is presented in Table 1. The initial step involves the conversion of the SMILES string of both solute and solvent into a graph structure using the RDKit package [20]. This package is employed not only for graph creation but also for the computation of atom and bond features for each graph. The selection of features was restricted to those computable in RDKit to mitigate the computational expenses associated with performing quantum mechanics calculations for the entire dataset. In order to standardize the lengths of the bond, atom, and global feature vectors, a linear transformation is applied to each vector before the commencement of the message-passing steps.

**Table 1.** Atoms (nodes), bonds (edges), and global features for molecular representation

Atomic features (V)	Bond features (E)	Global features (U)
Atomic species	Bond type	Total no. of atoms
No. of bonds	Conjugated status	Total no. of bonds
No. of bonded H atoms	Ring size	Molecular weight
Ring status	Stereo-chemistry	–
Valence	–	–
Aromatic status	–	–
Hybridization type	–	–
Donor status	–	–
Acceptor status	–	–
Partial charge	–	–

### Experimental setup

#### Datasets

This study utilizes the CombiSolv-Exp database, compiled by Vermeire and Green [11], which integrates experimental solvation data from multiple sources. Additionally, the FreeSolv database is incorporated, curated by Mobley and Guthrie [21], as well as the CompSol database from Moine et al. [22] and the Abraham dataset, collected by the Abraham group [23]. Furthermore, the Minnesota Solvation (MNSol) Database provides an extensive collection of 3,037 experimentally measured solvation free energies or transfer free energies [24]. These datasets collectively form a comprehensive benchmark for evaluating solvation energy predictions, as presented in Table 2.

**Table 2.** The dataset information

Dataset	Solvent	Solute	Pairs	Reference
FreeSolv	1	643	643	[21]
CompSol	259	442	3,548	[22]
Abraham	122	1,038	6,091	[23]
CompSolv-Exp	296	1,412	10,331	[11]
MNSol	92	790	3,037	[24]

#### Baselines

We compared MMGNN with some SOTA methods, and they could be classified into 2 categories. The first category of the models includes D-MPNN [11], SolvBert [25], SMD [26], Explainable GNN [6], GAT [27], GROVER [28], Uni-Mol [29], and Gem [30] that solely concatenate the molecular representations but ignore the interaction information between solvent and solute. The second category of the models includes methods like CIGIN [31] and CGIB [32], which implicitly considered the intermolecular interactions.

#### Experimental settings

MMGNN and MMHNN were trained using the Adam optimizer [33] with a learning rate decaying from 1×10^−4^ to 0.5, and a batch size of 50. The mean squared error was used as the training loss. Training was terminated if the validation error did not improve for 150 consecutive epochs or upon reaching the maximum limit of 1,000 epochs. All models were implemented in the PyTorch framework and trained on a Tesla A100 (40 GB) graphics processing unit.

#### Implementation details

The training is conducted with a batch size of 128. The model consists of a 4-layer GNN for message passing, with both node and edge embedding dimensions set to 300. For graph-level representation, we utilize the set2set pooling strategy. The feedforward neural network is configured with a feedforward dimension of 1,024 and a dropout rate of 0.1. A cosine annealing scheduler is used to adjust the learning rate during training. Early stopping is applied if the validation performance does not improve for 150 consecutive epochs, or training is terminated after a maximum of 1,000 epochs.

#### Evaluation metrics

Model performance was evaluated using mean absolute error (MAE) and root mean squared error (RMSE). The reported results include the mean and standard deviation, as summarized in the tables.

### Model performance

Similar to previous studies, a random split is employed to evaluate model performance. All methods were trained, validated, and tested on identical datasets using an 8:1:1 ratio in each test. The experimental results are presented in Table 3. Notably, MMHNN demonstrates superior predictive performance, consistently surpassing other baselines across the 3 test datasets. In terms of the MAE metric, MMHNN achieves an average reduction of 0.012 and 0.32 on the CompSol and CompSol-Exp datasets, respectively, compared to CGIB, the second-best model. Furthermore, performance exhibits a distinct ascending order across different interaction types, with models incorporating molecular interactions outperforming those that do not. This underscores the significance of explicitly modeling intermolecular interactions, as their comprehensive consideration substantially enhances predictive accuracy. The use of molecular merge graphs facilitates a more intrinsic representation of molecular interactions, rendering their relationships more explicit and pronounced.

**Table 3.** Test performance of different methods across 8 independent runs. Mean values are reported, with standard deviations shown in parentheses. (The best result in each column is underlined, while the top-performing baseline is marked with a superscript dagger.)

	MAE (↓)					RMSE (↓)				
Model	FreeSolv	CompSol	Abraham	CompSolv-Exp	MNSol	FreeSolv	CompSol	Abraham	CompSolv-Exp	MNSol
D-MPNN	0.684_(0.052)_	0.179_(0.013)_	0.454_(0.036)_	0.442_(0.022)_	0.459_(0.032)_	1.164_(0.055)_	0.343_(0.017)_	0.624_(0.024)_	0.672_(0.051)_	0.667_(0.017)_
Explainable GNN	0.724_(0.031)_	0.184_(0.012)_	0.486_(0.042)_	0.321_(0.013)_	0.396_(0.011)_	1.276_(0.045)_	0.367_(0.012)_	0.776_(0.035)_	0.404_(0.054)_	0.673_(0.024)_
SolvBERT	0.588_(0.034)_	0.167_(0.014)_	0.467_(0.034)_	0.382_(0.023)_	0.354_(0.021)_	1.021_(0.043)_	0.328_(0.020)_	0.652_(0.022)_	0.472_(0.041)_	0.623_(0.104)_
GAT	0.675_(0.033)_	0.187_(0.011)_	0.457_(0.043)_	0.970_(0.031)_	0.514_(0.043)_	1.185_(0.075)_	0.390_(0.012)_	0.726_(0.040)_	0.810_(0.101)_	0.812_(0.124)_
GROVER	0.623_(0.054)_	0.155_(0.022)_^†^	0.307_(0.035)_	0.382_(0.023)_	0.354_(0.024)_	1.015_(0.022)_	0.332_(0.016)_	0.475_(0.044)_	0.491_(0.053)_	0.672_(0.027)_
SMD	0.574_(0.036)_	0.162_(0.014)_	0.374_(0.024)_	0.633_(0.044)_	0.427_(0.034)_	1.113_(0.015)_	0.317_(0.011)_	0.516_(0.065)_	1.023_(0.152)_	0.682_(0.032)_
Uni-Mol	0.565_(0.038)_	0.164_(0.027)_	0.322_(0.071)_	0.214_(0.022)_	0.374_(0.021)_	1.002_(0.064)_	0.303_(0.020)_	0.602_(0.035)_	0.373_(0.043)_	0.657_(0.019)_
Gem	0.584_(0.041)_	0.174_(0.011)_	0.201_(0.065)_	0.253_(0.023)_	0.367_(0.025)_	1.131_(0.059)_	0.290_(0.019)_	0.641_(0.031)_	0.551_(0.023)_	0.675_(0.027)_
CIGIN	0.564_(0.057)_	0.164_(0.016)_	0.254_(0.010)_	0.241_(0.023)_	0.347_(0.023)_	0.910_(0.015)_	0.318_(0.020)_	0.404_(0.007)_	0.411_(0.032)_	0.644_(0.012)_
CGIB	0.531_(0.034)_^†^	0.156_(0.014)_	0.195_(0.005)_^†^	0.203_(0.033)_^†^	0.321_(0.017)_^†^	0.892_(0.022)_^†^	0.278_(0.018)_^†^	0.391_(0.006)_^†^	0.351_(0.031)_^†^	0.613_(0.023)_^†^
MMGNN	0.536_(0.030)_	0.146_(0.010)_	0.187_(0.008)_	0.171_(0.013)_	0.281_(0.011)_	0.902_(0.026)_	0.267_(0.012)_	0.385_(0.008)_	0.303_(0.033)_	0.607_(0.027)_
MMHNN	0.527_(0.028)_	0.144_(0.011)_	0.183_(0.007)_	0.172_(0.011)_	0.271_(0.011)_	0.894_(0.024)_	0.257_(0.010)_	0.379_(0.006)_	0.310_(0.027)_	0.585_(0.022)_

Further analysis was conducted on the degree of intermolecular information exchange, as presented in Table 4. Various γ values were examined to assess their impact on information transfer. A γ value of 0, which exclusively considers intramolecular interactions, results in the highest prediction error, as indicated by both MAE and RMSE metrics. Increasing the interaction level (γ ranging from 0 to 0.2) significantly improves predictive performance, leading to an 18.6% reduction in MAE. However, further increasing γ beyond 0.2 results in a performance decline, likely due to redundant and excessive information interfering with the model’s ability to capture essential patterns. These findings highlight the necessity of balancing intrinsic and intermolecular information to achieve optimal predictive performance.

**Table 4.** Test results of the MMHNN under different information interaction update rates and GNN backbones. Interaction coefficient is denoted as Δ. Bold indicates the best results and values are presented as mean (standard deviation).

Information Interaction	Δ and Backbones	MAE (↓)	RMSE (↓)
Intermolecular	γ = 1.0	0.192_(0.023)_	0.351_(0.042)_
	γ = 0.5	0.182_(0.013)_	0.342_(0.033)_
	γ = 0.2	**0.172**_(0.011)_	**0.310**_(0.027)_
	γ = 0.1	0.182_(0.013)_	0.358_(0.033)_
	γ = 0.0	0.204_(0.028)_	0.384_(0.055)_
	GAT	0.196_(0.012)_	0.383_(0.045)_
	GIN	0.213_(0.017)_	0.442_(0.035)_
	GCN	0.266_(0.026)_	0.452_(0.048)_
Intramolecular	GAT	0.202_(0.025)_	0.412_(0.037)_
	GCN	0.172_(0.011)_	0.310_(0.027)_
	GIN	0.183_(0.018)_	0.352_(0.026)_

Additionally, we analyzed the influence of different GNN backbones on molecular encoding strategies. MMHNN is a versatile framework that can be implemented with various GNN architectures, such as GAT, graph convolution network (GCN), and graph isomorphism network (GIN), to extract intermolecular information. In this evaluation, intramolecular interactions were modeled using the optimal γ value of 0.2, as determined in the previous experiments. Notably, the GCN-based model outperformed both GAT and GIN, achieving reductions of approximately 14.8% and 6.01% in MAE, respectively. Consequently, all subsequent experiments adopted the GCN framework for intramolecular message passing and a hyperparameter setting of 0.2 for intermolecular message passing.

### Explainability analysis

Four explainability methods, local mask-based, global mask-based, gradient-based, and GIB (detailed descriptions can be found in the Appendix D), are conducted separately in evaluations across various datasets and documented the optimal results for MMHNN. The results are presented in Table 5. Notably, the incorporation of explainability methods leads to a significant improvement in model performance. Among them, the GIB-based method consistently outperforms the other approaches, which means that the introduction of interpretability methods helps the model focus on important relationships, thereby improving model performance.

**Table 5.** Comparison of different explainability methods on various datasets. Underlined numbers are the best results and values are presented as mean (standard deviation).

Datasets	GIB-based	Global mask-based	Local mask-based	Gradient-based	Without explainability injection
Freesolv	0.527_(0.028)_	0.536_(0.010)_	0.547_(0.028)_	0.602_(0.010)_	0.651_(0.010)_
CompSol	0.144_(0.011)_	0.146_(0.010)_	0.154_(0.015)_	0.163_(0.017)_	0.175_(0.024)_
Abraham	0.183_(0.007)_	0.187_(0.010)_	0.189_(0.020)_	0.205_(0.026)_	0.328_(0.047)_
CompSol-Exp	0.172_(0.011)_	0.173_(0.013)_	0.171_(0.010)_	0.146_(0.010)_	0.208_(0.015)_
MNSol	0.269_(0.013)_	0.281_(0.011)_	0.305_(0.012)_	0.326_(0.024)_	0.342_(0.032)_

**Fig. 3.**
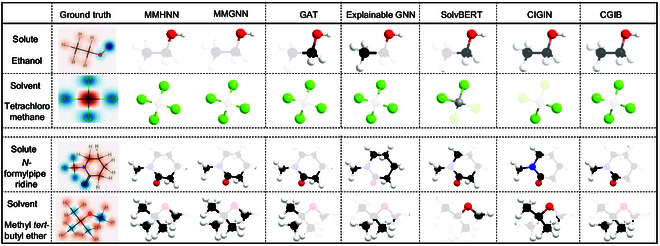
Core substructures identified by different methods. Atoms with values above the average score are retained based on atomic weighted scores. In the ground truth, the color transition from pink to blue indicates an increasing significance of the corresponding atom.

Further interpretability results are analyzed, and given the current lack of consensus on the key functional groups involved in the energy release process of solution solvents, we compiled a set of potentially important functional groups from relevant literature to serve as ground truth for validating algorithmic explainability [34,35]. We visualized pairwise solute–solvent interactions using interaction map weights. Specifically, we examined solvent–solute combinations that induce variations in solvation free energy ($\Delta G_{\text{solv}}$) from a chemical perspective by indexing atomic contributions based on attention weights. To enhance interpretability and provide an intuitive representation of functional group interactions, we aggregated the contribution scores for each atom rather than displaying individual atom–pairwise interactions. In the visualizations, solvation-enhancing interactions are highlighted in green, whereas solvation-inhibiting interactions are pink in Fig. 3, with color intensity indicating the relative strength of the effect.

It is evident that the significant substructures identified by MMHNN and MMGNN align closely with the ground-truth annotations, likely due to class-specific patterns captured by the explanatory subnetwork. In contrast, baseline explainability methods occasionally highlight edges that do not correspond to ground-truth annotations. This observation validates MMHNN’s capability to capture chemically meaningful substructure interactions, demonstrating its ability to extract implicit scientific knowledge from solute–solvent molecular interactions.

Compared to the substructures identified by baseline explainers, the subgraphs generated by MMHNN and MMGNN exhibit enhanced connectivity, a property attributed to their alternating and iterative selection processes that is absent in baseline explainers. For specific molecular nodes (e.g., N and O), certain neighboring nodes can introduce spurious signals, potentially leading to misleading explanations. MMGNN and MMHNN are explicitly designed to mitigate such issues, ensuring that the extracted explanations remain robust and interference-free. In contrast, baseline methods may include these confounding nodes, further underscoring the reliability and interpretability of MMHNN.

### Generalization test on solvents

**Fig. 4.**
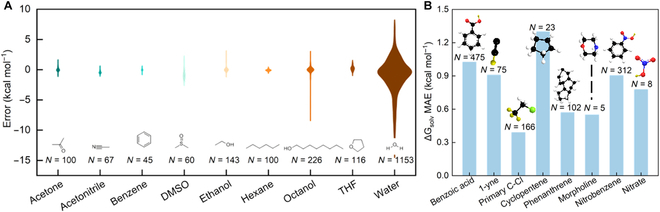
(A) Violin plots of the MAE ($\Delta G_{\text{pred}}-\Delta G_{\text{true}}$) for various solvents including acetone, acetonitrile, benzene, and DMSO. (B) Results of the scaffold holdout test.

To evaluate the model’s generalizability, a solvent holdout test (Fig. 4A) and a scaffold holdout test (Fig. 4B) are conducted by excluding specific solvents and scaffolds from the training and validation sets. MMHNN outperforms other models and has superior results on a variety of solvents, such as benzene (0.170 kcal mol^–1^), ethanol (0.23 kcal mol^–1^), hexane (0.12 kcal mol^–1^), and acetone (0.225 kcal mol^–1^), as shown in Table 6. However, the water solvent has the higher error compared to others. This difference could be intricately connected to factors such as the molecule’s molar mass and element type.

**Table 6.** Cross-test results for various methods for solvents holdout test. Underlined numbers are the best results. Values are presented as mean (standard deviation).

	MAE (↓)					RMSE (↓)				
Solvent	GAT	Explainable GNN	CGIB	**MMGNN**	**MMHNN**	GAT	Explainable GNN	CGIB	**MMGNN**	**MMHNN**
Acetone	0.301_(0.032)_	0.284_(0.023)_	0.252_(0.024)_	0.231_(0.012)_	0.225_(0.010)_	0.372_(0.022)_	0.355_(0.021)_	0.342_(0.024)_	0.331_(0.022)_	0.327_(0.020)_
Acetonitrile	0.592_(0.051)_	0.482_(0.023)_	0.454_(0.023)_	0.425_(0.030)_	0.421_(0.027)_	0.532_(0.051)_	0.484_(0.041)_	0.477_(0.040)_	0.462_(0.041)_	0.460_(0.038)_
Benzene	0.233_(0.013)_	0.241_(0.024)_	0.202_(0.014)_	0.174_(0.013)_	0.170_(0.010)_	0.455_(0.040)_	0.531_(0.104)_	0.278_(0.010)_	0.181_(0.0217)_	0.179_(0.015)_
DMSO	1.217_(0.164)_	0.977_(0.074)_	0.955_(0.483)_	0.432_(0.184)_	0.430_(0.194)_	1.121_(0.151)_	1.050_(0.071)_	1.074_(0.091)_	1.053_(0.071)_	1.050_(0.068)_
Ethanol	0.291_(0.024)_	0.262_(0.021)_	0.253_(0.022)_	0.231_(0.018)_	0.230_(0.016)_	0.532_(0.034)_	0.513_(0.052)_	0.435_(0.051)_	0.371_(0.023)_	0.371_(0.023)_
Octanol	0.452_(0.023)_	0.461_(0.045)_	0.374_(0.028)_	0.321_(0.024)_	0.319_(0.023)_	0.842_(0.034)_	0.854_(0.042)_	0.827_(0.031)_	0.825_(0.031)_	0.823_(0.030)_
THF	0.491_(0.032)_	0.453_(0.024)_	0.443_(0.032)_	0.432_(0.024)_	0.431_(0.020)_	0.512_(0.041)_	0.494_(0.042)_	0.476_(0.034)_	0.462_(0.032)_	0.460_(0.030)_
Water	2.584_(0.045)_	2.324_(0.037)_	2.117_(0.043)_	2.105_(0.052)_	2.100_(0.049)_	3.804_(0.119)_	3.204_(0.120)_	3.197_(0.104)_	3.162_(0.107)_	3.160_(0.100)_
Hexane	0.232_(0.014)_	0.498_(0.015)_	0.143_(0.015)_	0.124_(0.015)_	0.123_(0.014)_	0.217_(0.012)_	0.472_(0.031)_	0.203_(0.011)_	0.174_(0.012)_	0.174_(0.012)_

**Fig. 5.**
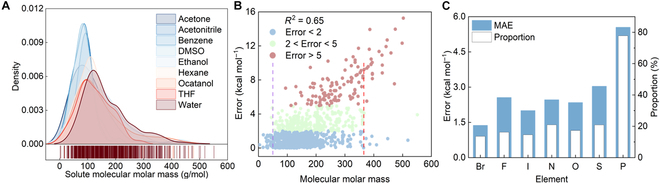
(A) The test solute molecular molar mass distributions in various solvents. (B) The relationship between molar mass of different solutes and prediction error in water solvent. (C) The element holdout test in water solvents and proportion of test molecules containing such elements in datasets.

The distribution of solute molar masses in each solvent, illustrated in Fig. 5A, provides a possible explanation. Most $\Delta G_{\text{solv}}$ data for polyatomic molecules are specifically measured in water, primarily as hydration Gibbs free energies. Molecular molar masses greater than 350 are predominantly present in aqueous solvents, constituting the test dataset for MMHNN. As a result, the model exhibits poor predictive performance due to the limited learning instances for these molecules. Clearly, octanol and water exhibit higher prediction errors compared to other solvents. This phenomenon is primarily attributed to the uneven distribution of solute molecules in the dataset. In particular, we found that the proportion of molecules with a molecular weight greater than 200 is higher in both octanol and water. This trend becomes even more pronounced for molecules exceeding 350 Da, compared to solvents such as ethanol (Fig. A1). This is reflected in the considerably higher test MAE for water solvent in Fig. 5A compared to other solvents.

Besides, we conducted an analysis of the relationship between the prediction errors of the model and the molar mass, and the number of atoms in the tested molecules in water solvent in Fig. 5B. Firstly, we could find that the model has excellent prediction results for molecules with a molar mass of less than 50, while the prediction error of the MMHNN model exhibits a high correlation with the molecular molar mass, with an *R*^2^ value of 0.65. This result suggests that the prediction error of the model may increase for larger molar mass molecules. However, a clear stratification phenomenon is observed in the predictive performance of MMHNN for molecules with molar masses between 50 and 350 in Fig. 5B, revealing a similar trend. This means that other factors may also play a significant role in affecting the model’s performance in this molar mass range.

In addition, we analyzed the impact of elements, as the water solvent may contain more solutes with other elements besides C and H. We show the results of removing the test elements contained in different solute molecules in the water solvent system and the proportion of such elements in the total dataset in Fig. 5C. Clearly, the MMHNN model exhibits the poorest generalization performance for the element P, with an error of 5.55. However, it is worth noting that approximately 77.8% of P-containing molecules are found in aqueous solvent systems. Therefore, in the solvent holdout experiment, there are only 4 instances of P-containing molecules in both the training and validation sets. This limited representation in the data may be another contributing factor to the poor predictive performance of the model for aqueous solvents.

### Generalization test on solute

To evaluate the model’s generalization capabilities on a new category of solute molecules, we performed a scaffold-based split by assigning all solutes containing one or more functional groups or molecular scaffolds according to the MoleculeNet benchmark [28,36,37]. The prediction results for different molecular scaffolds vary significantly across various solvent systems (in Table 7). Among the 8 solvent systems tested, the water solvent system has the largest prediction error, which may be attributed to the complexity of the system and the presence of molecules containing a variety of elements such as phosphorus in the R group. The cyclopentene scaffolds had the worst predicted result in the octanol system, which was significantly worse than other molecular skeleton results. This difference might be attributed to the nonpolar nature of the cyclopentene scaffold as mentioned above. In summary, we meticulously examined and tested the generalization capabilities of MMHNN, providing a solid foundation for the comprehensive assessment of our model’s performance.

**Table 7.** Test results (MAE and RMSE results) for various scaffolds under different solvent conditions (“-” indicates that molecules with this scaffold do not exist in the solvent). Values are presented as mean (standard deviation).

MAE (↓)									
Solvent/Solute	Acetone	Acetonitrile	Benzene	DMSO	Ethanol	Hexane	Octanol	THF	Water
Benzoic acid	0.27_(0.02)_	-	-	-	0.27_(0.01)_	-	0.13_(0.01)_	0.25_(0.01)_	2.32_(0.05)_
1-yne	0.12_(0.01)_	-	-	0.73_(0.12)_	-	0.21_(0.01)_	-	-	2.29_(0.05)_
Primary C-Cl	0.21_(0.01)_	-	0.13_(0.01)_	-	0.40_(0.03)_	0.16_(0.01)_	0.10_(0.01)_	0.28_(0.02)_	0.68_(0.03)_
Cyclopentene	-	-	-	0.67_(0.10)_	-	-	0.39_(0.08)_	-	1.77_(0.04)_
Phenanthrene	0.13_(0.01)_	-	-	-	0.07_(0.01)_	0.10_(0.01)_	0.12_(0.01)_	0.41_(0.03)_	2.16_(0.07)_
Morpholine	-	-	-	-	-	-	-	-	2.32_(0.08)_
Nitrobenzene	0.32_(0.03)_	0.25_(0.02)_	-	-	0.12_(0.01)_	0.12_(0.01)_	0.17_(0.01)_	0.29_(0.02)_	3.63_(0.12)_
Nitrate	-	-	-	-	-	-	-	-	1.14_(0.03)_
Total	0.23_(0.02)_	0.42_(0.03)_	0.17_(0.01)_	0.95_(0.18)_	0.23_(0.02)_	0.12_(0.01)_	0.32_(0.02)_	0.43_(0.03)_	2.10_(0.05)_
RMSE (↓)
	Acetone	Acetonitrile	Benzene	DMSO	Ethanol	Hexane	Octanol	THF	Water
Benzoic acid	0.34_(0.02)_	-	-	-	0.69_(0.05)_	-	0.21_(0.01)_	0.31_(0.02)_	3.00_(0.12)_
1-yne	0.14_(0.01)_	-	-	0.76_(0.06)_	-	0.21_(0.01)_	-	-	2.52_(0.09)_
Primary C-Cl	0.22_(0.01)_	-	0.13_(0.01)_	-	0.40_(0.02)_	0.20_(0.01)_	0.13_(0.01)_	0.37_(0.03)_	0.89_(0.07)_
Cyclopentene	-	-	-	0.67_(0.06)_	-	-	4.50_(0.12)_	-	2.01_(0.07)_
Phenanthrene	0.17_(0.01)_	-	-	-	0.08_(0.01)_	0.11_(0.01)_	0.13_(0.01)_	0.45_(0.03)_	2.34_(0.10)_
Morpholine	-	-	-	-	-	-	-	-	2.39_(0.10)_
Nitrobenzene	0.32_(0.03)_	0.25_(0.02)_	-	-	0.15_(0.01)_	0.15_(0.01)_	0.27_(0.02)_	0.32_(0.03)_	4.74_(0.13)_
Nitrate	-	-	-	-	-	-	-	-	1.24_(0.05)_
Total	0.33_(0.02)_	0.46_(0.04)_	0.18_(0.01)_	1.07_(0.09)_	0.37_(0.02)_	0.17_(0.01)_	0.82_(0.03)_	0.46_(0.03)_	3.16_(0.10)_

As shown in Fig. 4B, we considered 8 scaffolds including benzoic acid, 1-yne, primary C-Cl, cyclopentene, phenanthrene, morpholine, nitrobenzene, and nitrate as the test dataset. The remaining solutes were utilized for the training and validation datasets, ensuring that the solute compounds from the test set did not appear in the training or validation sets, and vice versa. This method of split is analogous to the commonly used Murcko scaffold splits in machine learning models for drug discovery. The scaffolds were chosen from a subgroup or subgraph as in previous studies [4].

In terms of predictive performance, heterocyclic and polycyclic molecules generally yield lower prediction errors compared to monocyclic counterparts, a trend that is also observed in the MMGNN model. For example, phenanthrene and morpholine—representative polycyclic and heterocyclic compounds—exhibit predictivity values of 0.57 and 0.56, respectively, whereas the monocyclic compound cyclopentene shows a significantly higher prediction error (1.31). We hypothesize that this discrepancy arises from the distinct physicochemical properties of these molecular classes: monocyclic molecules are more likely to be nonpolar, while polycyclic and heterocyclic compounds often possess polar functional groups. The presence of polar moieties, such as 1-yne and nitrate, appears to facilitate model learning, as these features contribute to more distinguishable spectral or structural signatures.

Overall, the MMHNN model exhibits strong generalization capability across diverse molecular scaffolds. Nonetheless, its predictive accuracy may be moderately reduced when extrapolating to unseen, nonpolar scaffolds that lack informative or distinctive features.

### Complex system testing

**Fig. 6.**
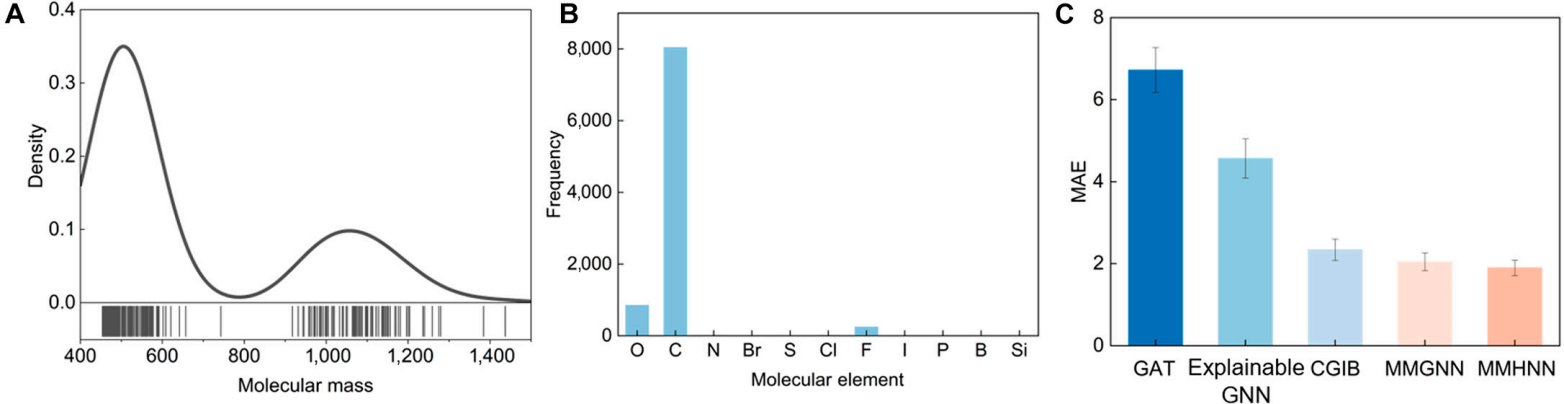
(A) Molecular weight distribution of the selected complex molecular pairs. (B) Elemental composition of the selected molecules. (C) Model performance on complex molecular systems.

Furthermore, to evaluate the model’s performance on complex molecular systems, we selected the top 300 molecular pairs with the highest relative molecular weights from the dataset. These samples represent larger and more structurally intricate molecules. As shown in Fig. 6A, the selected pairs exhibit significantly higher molecular weights compared to the overall distribution in the 5 benchmark datasets (see Fig. A1). Elemental analysis of these complex molecules, shown in Fig. 6B, reveals a concentration around C, O, and F elements, with a notably lower proportion of nitrogen compared to the full dataset. This suggests that complex molecules in the current dataset tend to be carbon-rich organic compounds rather than biomolecules. Performance results are reported in Fig. 6C, where MMHNN achieves superior predictive accuracy. The model significantly outperforms baseline methods such as GAT, Explainable GNN, and CGIB. This demonstrates the advantage of the merged-graph strategy in modeling complex molecular systems, where atomic interactions are highly intricate and difficult to quantify. By assigning adaptive weights to relational edges, the fused graph better captures subtle interatomic dependencies. Compared to MMGNN, our model further reduces prediction error by approximately 7.16%. With the improved core substructure extraction mechanism, it more precisely identifies key atomic relationships, enabling accurate and robust predictions in challenging molecular scenarios.

### Complexity analysis

**Fig. 7.**
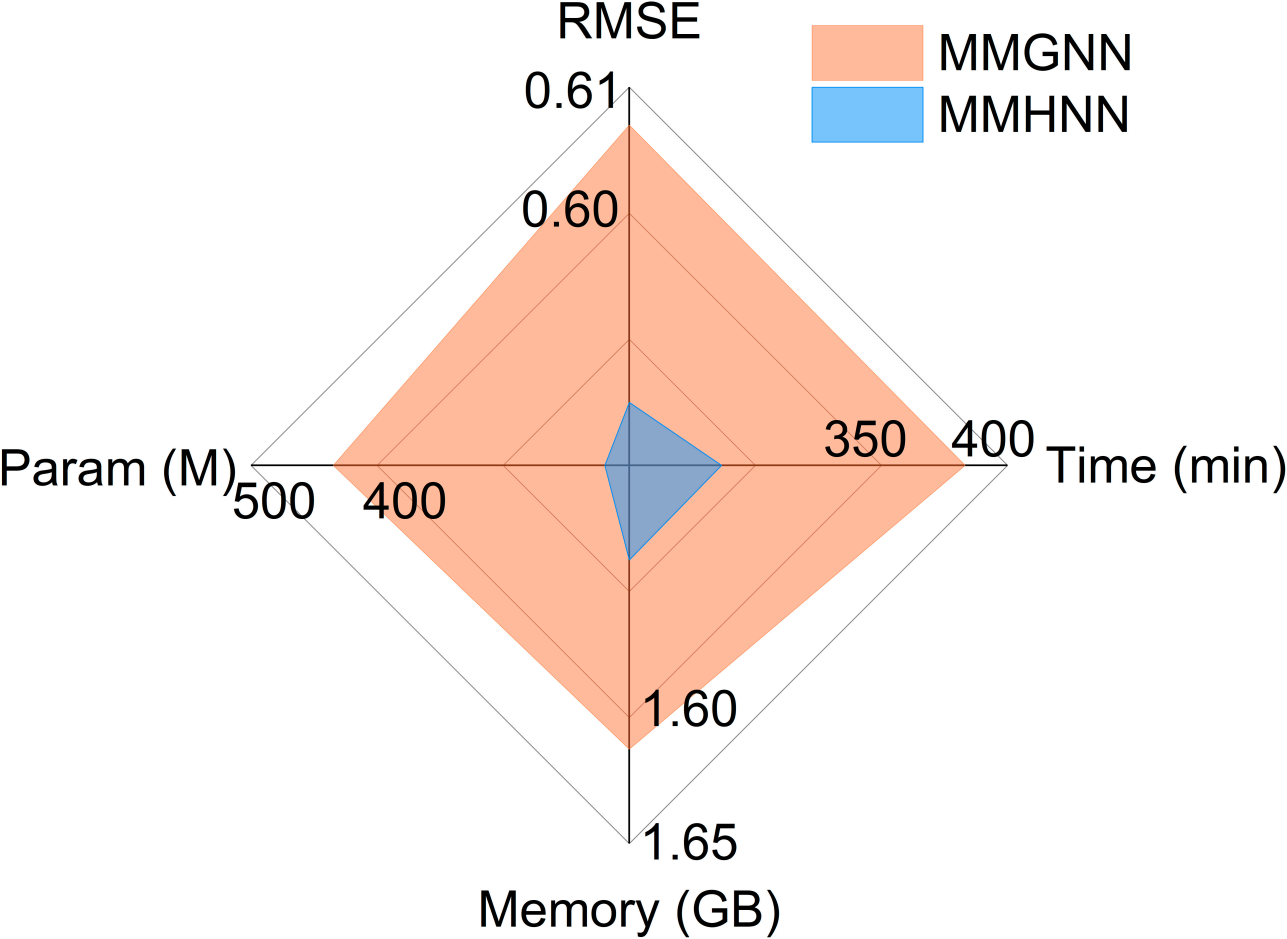
Comparison of resource consumption between MMGNN and MMHNN.

Constructing relationship edges in pairs, as previously done, has proven to be an effective strategy for predicting properties between molecular pairs. However, this approach significantly increases graph complexity, particularly for large, intricate molecules, due to the substantial rise in degrees of freedom. This leads to higher computational resource demands and increased time consumption. Motivated by the superior performance of the merged graph in molecular relationship learning, we are actively exploring advanced methods and techniques to reduce graph complexity. Currently, leveraging subgraph sets as hypernodes to construct a hypergraph is a promising approach. This method effectively reduces the complexity of the merged graph while maintaining the accuracy of molecular relationship learning as shown in Fig. 7. This design significantly reduces computational cost: model parameters decrease by approximately 90%, training time is reduced by 34%, and memory consumption drops by 6.25%, while predictive performance improves, with RMSE reduced by 3.62%. These results demonstrate that our approach not only simplifies the merged graph structure but also preserves—if not enhances—the accuracy of molecular relationship prediction.

### Sensitivity analysis

We analyze the influence of the hyperparameter β (ranging from 0 to 0.1) on subgraph extraction, which controls the trade-off between prediction accuracy and information compression, as defined in Eq. 25. When β=0, the model retains the full input graph without enforcing compression, leading to suboptimal performance due to the lack of structural abstraction. As β increases, performance initially improves as the model better identifies core substructures. However, beyond a certain threshold, further increasing β causes performance degradation. In particular, setting β=0.1 results in a significant drop, likely due to excessive compression that removes critical input features. These results are summarized in Table 8.

**Table 8.** Sensitivity analysis for β (RMSE indicator). Values are presented as mean (standard deviation).

	FreeSolv dataset				
2–6 β	0	1E−5	1E−4	1E−3	0.1
Result	0.882_(0.020)_	0.883_(0.021)_	0.879_(0.017)_	0.885_(0.024)_	1.164_(0.037)_

## Conclusion

In this study, we introduce MMHNN, a molecular graph neural network designed to predict solvation free energy. MMHNN leverages the complete connectivity between atoms within solute and solvent molecules, facilitating effective information transfer and enhancing model interpretability. Our experimental results demonstrate that MMHNN achieves superior performance across diverse datasets compared to existing methods, underscoring the importance of balancing intrinsic molecular properties and intermolecular interactions. The model also exhibits strong generalization capabilities, outperforming baselines in tasks such as solvent holdout and solute scaffold split. Moreover, MMHNN further enhances model performance while significantly reducing the complexity of the merged graph. The transparent interpretability of our model aligns well with established chemical principles, offering insights that resonate with experimental chemists. Additionally, a deeper understanding of molecular interactions provides valuable perspectives for assessing drug compound stability and guiding the development of novel synthetic pathways and catalytic processes.

## Methods

In this section, we provide detailed descriptions of the implementation of each module.

### Molecular graph representation

The molecular structure can be represented as a topological graph, where atoms correspond to nodes and bonds to edges [38,39]. Formally, the graph is defined as:

$$G=\left\{E,V,U\right\}$$ (1)

where V and E denote the sets of nodes and edges, respectively, and U represents the global molecular features. In this formulation, all bonds within a molecule are explicitly enumerated. The edge set E consists of triplets $\left(e_{k},p_{k},q_{k}\right)$, where $e_{k}$ represents the edge features, while $p_{k}$ and $q_{k}$ denote the 2 atoms connected by bond $e_{k}$. The total number of bonds in the molecule is denoted as $N^{e}$:

$$E={\left(e_{k},p_{k},q_{k}\right)}_{k=1}^{N^{e}},\;V={\left\{v_{i}\right\}}_{i=1}^{N^{v}}.$$ (2)

Similarly, the node set V enumerates all atoms in the molecule, where $v_{i}$ represents the atomic feature vector for atom i (e.g., atomic species, valence, etc.), and $N^{v}$ is the total number of atoms. With these definitions, the molecular graph is fully specified.

For solute–solvent interactions, each dataset entry consists of a pair of molecular graphs, $G_{1}$ and $G_{2}$, representing the solute and solvent molecules, respectively, along with their corresponding scalar label, such as the Gibbs free energy of solvation Y, where $Y\in \left(-\infty ,\infty \right)$.

### Hypergraph construction

In order to complete the construction of the hypergraph and further realize the widely intermolecular message passing, we firstly need to complete the extraction of the substructure. Correspondingly, the subgraph set $\left\{G_{\mathrm{sub}1}^{1},\ldots ,G_{sub1}^{c_{1}}\right\}$ and $\left\{G_{\mathrm{sub}2}^{1},\ldots ,G_{sub2}^{c_{2}}\right\}$ from $G_{1}$ and $G_{2}$ will be obtained through the principal subgraph algorithm [40], where $c_{1}\;and\;c_{2}$ represents the total number of subgraphs in G1 and G2 respectively. The extraction process is initialized by constructing the vocabulary V with all unique atoms, which are considered as subgraphs with a single node. This initialization can be represented as:

$$V=\left\{v|v\in \underset{G\in D}{\cup }G\right\},$$ (3)

where D denotes the dataset containing all graphs.

During the merging phase, each pair of neighboring fragments F and $F^{\prime }$ in the current vocabulary is concatenated to create a new fragment. For each fragment pair $\left(F,F^{\prime }\right)$, the merging operation can be described as:

$$F\cup F^{\prime }\to F^{\prime \prime },$$ (4)

where $F^{\prime \prime }$ is the new concatenated fragment. This operation is repeated iteratively.

The frequency of each concatenated subgraph is then counted, and the most frequent one is added to the vocabulary V. This updating process continues until the vocabulary size reaches a predefined number N. The frequency update for a subgraph S can be expressed as:

$$\text{frequency}\left(G_{\mathrm{sub}}\right)=\sum_{G_{i}\in D}I\left(G_{\mathrm{sub}}\subseteq G_{i}\right),$$ (5)

where I represents an indicator function that takes the value 1 if $G_{\mathrm{sub}}$ is a subgraph of $G_{i}$ and 0 otherwise.

Through these steps, the subgraph sets $\left\{G_{\mathrm{sub}1}^{1},\ldots ,G_{\mathrm{sub}1}^{c_{1}}\right\}$ and $\left\{G_{\mathrm{sub}2}^{1},\ldots ,G_{\mathrm{sub}2}^{c_{2}}\right\}$ are effectively extracted from $G_{1}$ and $G_{2}$ using the principal subgraph algorithm. This subgraph extraction method is particularly representative for solute–solvent datasets because both solute and solvent molecules commonly share similar substructure types.

Once the subgraph sets are obtained, the original graphs $G_{1}$ and $G_{2}$ can be transformed into hypergraphs, where each subgraph is treated as a hypernode. This transformation allows the complex structure of the original molecular graphs to be represented more compactly. The hypergraphs $H_{1}$ and $H_{2}$ derived from $G_{1}$ and $G_{2}$ can be defined as:

$$H_{1}=\left\{G_{\mathrm{sub}1}^{1},G_{\mathrm{sub}1}^{2},\ldots ,G_{\mathrm{sub}1}^{c_{1}}\right\},$$ (6)

$$H_{2}=\left\{G_{\mathrm{sub}2}^{1},G_{\mathrm{sub}2}^{2},\ldots ,G_{\mathrm{sub}2}^{c_{2}}\right\}.$$ (7)

In these hypergraphs, each hypernode represents a principal subgraph extracted from the original graph. Note that in these hypergraphs, edges do not carry meaningful information. During the intermolecular interaction process, we utilize hypernodes within the hypergraph for interaction.

### Intramolecular message passing process

For the encoded solute–solvent molecular graph, we will perform L rounds of message passing, in which intramolecular message passing and intermolecular message passing mechanisms are performed alternately. We begin by performing message passing independently within each molecule—namely, the solute and solvent. During this intramolecular phase, each bond feature $e_{k}$ is updated to a refined representation $e_{\mathrm{ij}}^{\prime }$ by integrating the initial bond attributes, the features of the connected atoms $v_{i}$ and $v_{j}$, and the global molecular descriptor u. Concurrently, the atomic representation $v_{i}$ for each atom i is updated to a new vector $v_{i}^{\prime }$.

$$e_{\mathrm{ij}}^{\prime }=e_{\mathrm{ij}}+\tau \left[\mathrm{FC}\left(v_{i}+v_{j}\right)+\mathrm{FC}\left(e_{\mathrm{ij}}\right)+\mathrm{FC}\left(u\right)\right],$$ (8)

$${\hat{e}}_{\mathrm{ij}}=\frac{\sigma \left(e_{\mathrm{ij}}^{\prime }\right)}{\sum_{j^{\prime }\in N_{i}}\sigma \left(e_{ij^{\prime }}^{\prime }\right)+\epsilon },$$ (9)

$$v_{i}^{\prime }=v_{i}+\tau \left[\mathrm{FC}\left(v_{i}+\sum_{j\in N_{i}}{\hat{e}}_{\mathrm{ij}}⊙\mathrm{FC}\left(v_{j}\right)\right)+\mathrm{FC}\left(u\right)\right],$$ (10)

where $v_{i}^{\prime }$ and $e_{\mathrm{ij}}^{\prime }$ are the updated node vectors and edge vectors. τ is the LeakyReLU activation function [41]. *FC* is the fully connected layer. ⊙ denotes the Hadamard product. ϵ is a fixed constant.

### Intermolecular message passing process

Then, we describe the intermolecular messaging stage, which facilitates the exchange of information between hypernode $\left\{G_{\mathrm{sub}1}^{1},\ldots ,G_{\mathrm{sub}1}^{c_{1}}\right\}$ and $\left\{G_{\mathrm{sub}2}^{1},\ldots ,G_{\mathrm{sub}2}^{c_{2}}\right\}$ of hypergraphs $H_{1}$ and $H_{2}$. This stage involves several key steps as follows.

First, for each hypernode $G_{\mathrm{sub}1}^{i}$ from hypergraph $H_{1}$, we apply the Pooling operation to aggregate the features of its nodes $V_{\mathrm{sub}1}^{i}$. This operation is defined as:

$$H_{\mathrm{sub}1}^{i}=\text{Pooling}\left(V_{\mathrm{sub}1}^{i}\right),$$ (11)

where $H_{\mathrm{sub}1}^{i}$ represents the feature vector for hypernode $G_{\mathrm{sub}1}^{i}$.

Similarly, for each hypernode $G_{\mathrm{sub}2}^{i}$ from hypergraph $H_{2}$, we perform the same Pooling operation:

$$H_{\mathrm{sub}2}^{i}=\text{Pooling}\left(V_{\mathrm{sub}2}^{i}\right),$$ (12)

where $H_{\mathrm{sub}2}^{i}$ represents the feature vector for hypernode $G_{\mathrm{sub}2}^{i}$.

Next, we compute the attention coefficients between each pair of hypernode feature vectors $\left(H_{\mathrm{sub}1}^{i},H_{\mathrm{sub}2}^{j}\right)$. These coefficients are calculated using the softmax function applied to the dot product of the feature vectors:

$$\alpha_{\mathrm{ij}}=\frac{\text{exp}\left(H_{\mathrm{sub}1}^{i}\cdot H_{\mathrm{sub}2}^{j}\right)}{\sum_{k}\text{exp}\left(H_{\mathrm{sub}1}^{i}\cdot H_{\mathrm{sub}2}^{k}\right)}.$$ (13)

Using these attention coefficients, we update the feature vectors of the hypernodes by incorporating information from their corresponding pairs. The updated feature vectors are given by:

$$H_{\mathrm{sub}1}^{i}=\sum_{j}\alpha_{\mathrm{ij}}H_{\mathrm{sub}2}^{j},\;H_{\mathrm{sub}2}^{j}=\sum_{i}\alpha_{\mathrm{ij}}H_{\mathrm{sub}1}^{i}.$$ (14)

Finally, we update the features of each node in the original graphs $G_{1}$ and $G_{2}$ by incorporating the information from their corresponding hypernode feature vectors. For each node v in $V_{1}^{\left(k\right)}$, the updated feature vector is computed as:

$$\begin{array}{c} V_{1}^{\left(k\right)}\left(v\right)=\left(1-\gamma \right)V_{1}^{\left(k\right)}\left(v\right)+\gamma H_{\mathrm{sub}1}^{i},\text{where}\;v\in G_{\mathrm{sub}1}^{i}. \end{array}$$ (15)

Similarly, for each node v in $V_{2}^{\left(k\right)}$, the updated feature vector is given by:

$$\begin{array}{c} V_{2}^{\left(k\right)}\left(v\right)=\left(1-\gamma \right)V_{2}^{\left(k\right)}\left(v\right)+\gamma H_{\mathrm{sub}2}^{j},\text{where}\;v\in G_{\mathrm{sub}2}^{j}. \end{array}$$ (16)

where γ is a hyperparameter that controls the magnitude of message updates. This intermolecular message passing process effectively facilitates information transfer between hypergraphs, capturing the comprehensive features of solute–solvent molecular combinations, and ultimately enhancing the representation of the original molecular graphs $G_{1}$ and $G_{2}$.

### Core hypernode extraction based on GIB

The GIB principle [42–44] is as follows:

$$G_{\mathrm{IB}}=\underset{G_{\mathrm{sub}}\in S}{\text{arg min}}-I\left(Y;G_{\mathrm{sub}}\right)+\beta I\left(G;G_{\mathrm{sub}}\right).$$ (17)

Intuitively, $G_{\mathrm{IB}}$ is the core subgraph of G, which discards information from G by minimizing the mutual information $I\left(G;G_{\mathrm{sub}}\right)$, while preserving target-relevant information by maximizing the mutual information $I\left(Y;G_{\mathrm{sub}}\right)$.

To detect the core reaction structure in hypergraphs $H_{1}$ and $H_{2}$, we optimize the model with the objective function defined in Eq. 17 as follows:

$$\begin{array}{c} H_{\mathrm{IB}1},H_{\mathrm{IB}2}=\\ \;\underset{H_{\mathrm{IB}1},H_{\mathrm{IB}2}}{\text{arg min}}-I\left(Y;H_{\mathrm{IB}1},H_{\mathrm{IB}2}\right)+\beta \left(I\left(H_{1};H_{\mathrm{IB}1}\right)+I(H_{2};H_{\mathrm{IB}2})\right), \end{array}$$ (18)

In the following sections, we provide the proof of upper bound of each term, which should be minimized during training.

#### Extract target-oriented information

To minimize$-I\left(Y;H_{\mathrm{IB}1},H_{\mathrm{IB}2}\right)$, we aim to calculate the upper bound of $-I\left(Y;H_{\mathrm{IB}1},H_{\mathrm{IB}2}\right)$. Given the hypergraph $H_{1}$, $H_{2}$, its label information Y, and the learned IB-graph $H_{\mathrm{IB}1}$, $H_{\mathrm{IB}2}$, we have:

$$\begin{array}{c} \begin{array}{c} \begin{array}{l} -I\left(Y;H_{\mathrm{IB}1},H_{\mathrm{IB}2}\right)\leq E_{Y;H_{\mathrm{IB}1},H_{\mathrm{IB}2}}\left[-\text{log}\;p_{\theta }\left(Y|H_{\mathrm{IB}1},H_{\mathrm{IB}2}\right)\right]≔L_{\mathrm{pre}}, \end{array} \end{array} \end{array}$$ (19)

where $p_{\theta }\left(Y|H_{\mathrm{IB}1},H_{\mathrm{IB}2}\right)$ is the variational approximation of $p\left(Y|H_{\mathrm{IB}1},H_{\mathrm{IB}2}\right)$. We model $p_{\theta }\left(Y|H_{\mathrm{IB}1},H_{\mathrm{IB}2}\right)$ as a predictor parametrized by θ. Thus, we can minimize the upper bound of $-I\left(Y;H_{\mathrm{IB}1},H_{\mathrm{IB}2}\right)$ by minimizing the model prediction loss $L_{\mathrm{pre}}\left(Y,H_{\mathrm{IB}1},H_{\mathrm{IB}2}\right)$ with cross entropy loss. The proof is given in Appendix B.

#### Optimizing the minimization of $H_{1}$

$\text{Minimizing}\;I\left(H_{1};H_{\mathrm{IB}1}\right)$, we aim to derive an upper bound of the mutual information term $I\left(H_{1};H_{\mathrm{IB}1}\right)$. We adopt a strategy by injecting noise into hypernode representations to suppress redundant information in $H_{1}$ [43]. Specifically, we introduce noise sampled from a parameterized distribution and probabilistically replace hypernode features with this noise. For each hypernode i, a transmission probability $p_{i}$ is learned via a fully connected (*FC*) layer applied to its embedding $H_{\mathrm{sub}1}^{i}$, followed by a sigmoid activation to ensure $p_{i}\in \left[0,1\right]$:

$$\begin{array}{l} p_{i}=\text{Sigmoid}\left(\mathrm{FC}\left(H_{\mathrm{sub}1}^{i}\right)\right). \end{array}$$ (20)

Then, the hypernode representation $H_{\mathrm{sub}1}^{i}$ is stochastically replaced with noise ϵ based on the learned $p_{i}$. The resulting representation $z_{i}$ is formulated as:

$$\begin{array}{l} z_{i}=\lambda_{i}H_{\mathrm{sub}1}^{i}+\left(1-\lambda_{i}\right)\epsilon , \end{array}$$ (21)

where $\lambda_{i}∼\text{Bernoulli}\left(p_{i}\right)$ denotes a binary sampling variable that determines whether the original representation is preserved or replaced with noise. This stochastic gating mechanism enables fine-grained control over the information flow from $H_{\mathrm{sub}1}^{i}$ to $z_{i}$. When $p_{i}=1$, all information is retained; when $p_{i}=0$, it is fully replaced by noise. To enable gradient-based optimization despite the discrete nature of $\lambda_{i}$, we adopt the concrete relaxation [45], defined as:

$$\begin{array}{l} \lambda_{i}=\text{Sigmoid}\left(\frac{1}{t}\text{log}\frac{p_{i}}{1-p_{i}}+\text{log}\frac{u}{1-u}\right), \end{array}$$ (22)

where t is the temperature parameter and $u∼\text{Uniform}\left(0,1\right)$. Another critical aspect of noise injection is the characterization of the injected noise. It is important that arbitrary noise can be detrimental to the semantic integrity of the input hypergraph, leading to predictions that deviate from the actual hypergraph properties. Conversely, appropriately selected noise can provide a variational upper bound to the overall objective. Therefore, the minimized upper bound of $I\left(H_{1};H_{\mathrm{IB}1}\right)$ is as follows:

$$\begin{array}{c} \begin{array}{c} I\left(H_{1};H_{\mathrm{IB}1}\right)\leq E_{H_{1}}[-\frac{1}{2}\text{log}\;A_{H_{1}}+\\ \frac{1}{2m_{H_{1}}}A_{H_{1}}+\frac{1}{2m_{H_{1}}}B_{H_{1}}^{2}]≔L_{{\mathrm{MI}}_{1}}\left(H_{\mathrm{IB}1,H_{1}}\right), \end{array} \end{array}$$ (23)

where $A_{H_{1}}=\sum_{j=1}^{m_{H_{1}}}{\left(1-\lambda_{j}\right)}^{2}$ and $B_{H_{1}}=\frac{\sum_{j=1}^{m_{H_{1}}}\lambda_{j}\left(H_{\mathrm{sub}1}^{j}-\mu_{H}\right)}{\sigma_{H}}$. A detailed proof is given in Appendix C.

#### Optimizing the minimization of $H_{2}$

$\text{In order to minimize}\;I\left(H_{2};H_{\mathrm{IB}2}\right)$, we aim to calculate the upper bound of $I\left(H_{2};H_{\mathrm{IB}2}\right)$. Similarly, we have:

$$\begin{array}{c} \begin{array}{c} I\left(H_{2};H_{\mathrm{IB}2}\right)\leq E_{H_{2}}[-\frac{1}{2}\text{log}\;A_{H_{2}}+\\ \frac{1}{2m_{H_{2}}}A_{H_{2}}+\frac{1}{2m_{H_{2}}}B_{H_{2}}^{2}]≔L_{{\mathrm{MI}}_{2}}\left(H_{2},H_{\mathrm{IB}2}\right). \end{array} \end{array}$$ (24)

### Loss function

In summary, our overall loss function is formulated by combining these individual components as follows, providing a comprehensive approach to optimizing our MMHNN for the intended molecular interaction tasks:

$$\begin{array}{c} L_{\mathrm{sum}}=L_{\mathrm{pre}}+\beta \left(L_{{\mathrm{MI}}_{1}}+L_{{\mathrm{MI}}_{2}}\right), \end{array}$$ (25)

where β is the trade-off parameter. $L_{\mathrm{pre}}$ is the binary cross entropy loss incorporating logits employed to mitigate prediction errors. $L_{{\mathrm{MI}}_{1}}$ and $L_{{\mathrm{MI}}_{2}}$ represent Kullback–Leibler divergence between extracted core subgraphs and random noise graphs, encouraging subgraph compression.

## Data Availability

All of the data and code could be found in https://github.com/invokerqwer/MMHNN.

## Supplementary Materials

Supplementary 1 The supplementary appendix provides the main theoretical proofs.
