# Supplementary material for: Molecular Merged Hypergraph Neural Network for Explainable Solvation Gibbs Free Energy Prediction
Source: Research (Wash D C). 2025 Aug 15;8:0740. doi: 10.34133/research.0740 (PMC12355008; doi:10.34133/research.0740)
Supplement: Supplementary 1 — The supplementary appendix provides the main theoretical proofs. [file research.0740.f1.pdf]

MMHNN: A Molecular Merged Hypergraph Neural Network for Explainable Solvation Free Energy Prediction

Wenjie Du[1,2], Shuai Zhang[2], Zhaohui Cai[3,4], Xuqiang Li[1,2], Zhiyuan Liu[5], Junfeng Fang[5,*], Jianmin Wang[6,*], and Yang Wang[1,2,*]

[1]Key Laboratory of Precision and Intelligent Chemistry, University of Science and Technology of China, Hefei, Anhui 230026, China
[2]Suzhou Institute for Advanced Research, University of Science and Technology of China, Suzhou, Jiangsu 215123, China.
[3]Suzhou Laboratory, Suzhou 215000, China.
[4]School of Advanced Technology, Xi'an Jiaotong-Liverpool University, Suzhou 215123, P.R. China.
[5]School of Computing, National University of Singapore, Singapore 117417.
[6]Department of Integrative Biotechnology, Yonsei University, Incheon 21983, Republic of Korea.
[*]Address correspondence to: jmwang113@hotmail.com; fangjf1997@gmail.com; angyan@ustc.edu.cn

A Related Work

In this section, we introduce Molecular Relational Learning and Gibbs Free Energies Prediction Methods.

A.1 Molecular Relational Learning

Generally, message passing in GNNs or graph convolutional networks (GCNs) refers to the utilization of trainable interaction layers to facilitate the exchange of information among atoms within a local neighborhood [1–3]. However, this approach compromise to capture the long-range effects that can arise between intermolecular such as charge transfer, polarization etc. This is attributed to the limited locality assumption of atomic environments, which usually employ a cut-off radius of 5-7 Å and may not adequately account for all intermolecular forces [4]. Behler and Parrinello are pioneers in modelling interatomic properties [5–7] by summing per-atom contributions in neural network predictions. Tensormol [8] employe Behler and Parrinello's approximation to accurately compute the dipole moment in water dimers as one water molecule rotates about the O-H bond, resulting in reasonable outcomes. This method is subsequently refined by integrating the atomic-pairwise into the neural network [9] which led to a reduction in the binding energy error by a factor of five. [10] leverages message passing neural networks and attention mechanism to encode the representation of atoms to predict the solvation free energy. [11] provide a molecular relational learning framework that predicts the interaction behavior based on graph information bottleneck theory. In brief, the routine method is summing overall the atoms, or fusing information after message passing process but a more rigorous way should be considering the molecular atoms or fragments and introducing atom-pair symmetry message passing functions [12].

A.2 Gibbs Free Energies Prediction Methods

Solvation free energies have been of great interest for many years and have spurred the development of numerous predictive methods [13]. These methods encompass a range of techniques, including molecular dynamics and quantum chemistry methods, as well as empirical or data-driven approaches. While quantum chemistry methods such as the SMx and COSMO(-RS) models [14, 15] are based on first-principles calculations of all promising relevant conformers of the solute and solvent molecules, they are often computationally expensive and labor-intensive. In contrast, empirical or data-driven methods could provide a faster alternative for predicting solvation properties. Recent studies have aimed at improving these models for predicting ΔG_{solv}. [16] utilized a transfer learning method to achieve a mean absolute error of 0.21 kcal/mol when predicting experimental results from a standard quantum computing dataset. similarly, [17] calculated water solvation Gibbs free energies of more than 100,000 organic compounds, and utilized a graph neural network (GNN) to predict these values achieving an error of 0.4 kcal/mol. Lim and Jung [18] and Pathak et al. [10] both utilized a GNN model to train on a dataset containing 5,597 measurements, and achieved a low mean absolute error (MAE) of 0.2 kcal/mol on their respective randomly divided test sets. In another approach, Kaycee et al. [19] incorporated additional atomic features and solvation-relevant parameters as the chemical intuition into the featurization process to enhance the interpretability.

Algorithm 1 MMHNN

Require: Input graphs $\mathcal{G}_1 = \{\mathcal{V}_1, \mathcal{E}_1, \mathcal{U}_1\}$ and $\mathcal{G}_2 = \{\mathcal{V}_2, \mathcal{E}_2, \mathcal{U}_2\}$, initial parameters $\theta^{(0)}$

  Preprocessing: Build the hypergraphs $\mathcal{H}_1 = \{\mathcal{G}^1_{sub1}, \mathcal{G}^2_{sub1}, \ldots, \mathcal{G}^{c_1}_{sub1}\}$, $\mathcal{H}_2 = \{\mathcal{G}^1_{sub2}, \mathcal{G}^2_{sub2}, \ldots, \mathcal{G}^{c_2}_{sub2}\}$ using frequent subgraph mining.

  Initialize: $\theta \leftarrow \theta^{(0)}$

  repeat

    Forward Pass:

    for $k = 1$ to 4 **do**

      Intramolecular messaging stage:

      for each bond (i, j) in $\mathcal{E}_1$ and $\mathcal{E}_2$ **do**

        Update edge features $e^{(k)}_{ij}$

      end for

      for each atom i in $\mathcal{V}_1$ and $\mathcal{V}_2$ **do**

        Update node features $v^{(k)}_i$

      end for

      Intermolecular messaging stage:

      for each hypernode $\mathcal{G}^i_{sub1}$ in hypergraph $\mathcal{H}_1$ **do**

        $\mathbf{H}^i_{sub1} \leftarrow \text{Pooling}(\mathcal{V}^i_{sub1})$

      end for

      for each hypernode $\mathcal{G}^i_{sub2}$ in hypergraph $\mathcal{H}_2$ **do**

        $\mathbf{H}^i_{sub2} \leftarrow \text{Pooling}(\mathcal{V}^i_{sub2})$

      end for

      for each pair $(\mathbf{H}^i_{sub1}, \mathbf{H}^j_{sub2})$ **do**

        $\alpha_{ij} \leftarrow \text{softmax}(\mathbf{H}^i_{sub1} \cdot \mathbf{H}^j_{sub2})$

        $\mathbf{H}^i_{sub1} \leftarrow \alpha_{ij}\mathbf{H}^j_{sub2}$

        $\mathbf{H}^j_{sub2} \leftarrow \alpha_{ij}\mathbf{H}^i_{sub1}$

      end for

      for each node v in $\mathcal{V}^{(k)}_1$ **do**

        $\mathcal{V}^{(k)}_1(v) = \gamma\mathcal{V}^{(k)}_1(v) + (1-\gamma)\mathbf{H}^i_{sub1} \quad \text{where} \quad v \in \mathcal{G}^i_{sub1}$

      end for

      for each node v in $\mathcal{V}^{(k)}_2$ **do**

        $\mathcal{V}^{(k)}_2(v) = \gamma\mathcal{V}^{(k)}_2(v) + (1-\gamma)\mathbf{H}^i_{sub2} \quad \text{where} \quad v \in \mathcal{G}^i_{sub2}$

      end for

    end for

    Core Hypernode Extraction:

    Optimize the objective function with GIB to extract core hypernodes $\mathcal{H}_{\text{IB1}}$ and $\mathcal{H}_{\text{IB2}}$ from hypergraphs $\mathcal{H}_1$ and $\mathcal{H}_2$:

$$\mathcal{H}_{\text{IB1}}, \mathcal{H}_{\text{IB2}} = \underset{\mathcal{H}_{\text{IB1}}, \mathcal{H}_{\text{IB2}}}{\arg\min}$$

    -I(Y; $\text{H}_{\text{IB1}}, \mathcal{H}_{\text{IB2}}) + \beta\left(I(\mathcal{H}_1; \mathcal{H}_{\text{IB1}}) + I(\mathcal{H}_2; \mathcal{H}_{\text{IB2}})\right)$

    Pooling:

    $\mathcal{H}_{\mathcal{G}_1} \leftarrow \mathcal{H}_{\text{IB1}}$

    $\mathcal{H}_{\mathcal{G}_2} \leftarrow \mathcal{H}_{\text{IB2}}$

    Concatenate:

    $\mathcal{H}_{\text{concat}} \leftarrow \text{concat}(\mathcal{H}_{\mathcal{G}_1}, \mathcal{H}_{\mathcal{G}_2})$

    Fully Connected Layer:

    $\hat{\mathbf{Y}} \leftarrow \mathbf{W}_{\text{fc}} \cdot \mathcal{H}_{\text{concat}} + \mathbf{b}_{\text{fc}}$

    Loss Calculation:

    Compute the combined loss:

$$\mathcal{L} = \mathcal{L}_{pre} + \beta\left(\mathcal{L}_{\text{MI}_1} + \mathcal{L}_{\text{MI}_2}\right)$$

    Backward Pass:

    Update parameters $\theta \leftarrow \theta - \eta\nabla_\theta\mathcal{L}$

  until convergence criterion is met

  return Final model parameters θ =0

B Proof of $\mathcal{L}_{\mathrm{MI}}$

Proof. In this proof process we do not distinguish $\mathcal{H}_1$ and $\mathcal{H}_2$. We first use a readout function to obtain the graph representation $z_{\mathcal{H}_{\text{IB}}}$ of the perturbed graph $\mathcal{H}_{\text{IB}}$. And we assume these is no information loss in this process. Therefore we have $I\left(z_{\mathcal{H}_{\text{IB}}};\mathcal{H}\right) \approx I\left(\mathcal{H}_{\text{IB}};\mathcal{H}\right)$. Now we bound $I\left(z_{\mathcal{H}_{\text{IB}}};\mathcal{H}\right)$ using variational approximation:

$$
\begin{aligned}
I\left(z_{\mathcal{H}_{\text{IB}}};\mathcal{H}\right) &= \iint p\left(z_{\mathcal{H}_{\text{IB}}},\mathcal{H}\right)\log\frac{p\left(z_{\mathcal{H}_{\text{IB}}}\mid\mathcal{H}\right)}{p\left(z_{\mathcal{H}_{\text{IB}}}\right)}\,\mathrm{d}z_{\mathcal{H}_{\text{IB}}}\,\mathrm{d}\mathcal{H} \\
&= \iint p\left(z_{\mathcal{H}_{\text{IB}}},\mathcal{H}\right)\log\frac{p\left(z_{\mathcal{H}_{\text{IB}}}\mid\mathcal{H}\right)}{q\left(z_{\mathcal{H}_{\text{IB}}}\right)}\,\mathrm{d}z_{\mathcal{H}_{\text{IB}}}\,\mathrm{d}\mathcal{H} \\
&\quad + \iint p\left(z_{\mathcal{H}_{\text{IB}}},\mathcal{H}\right)\log\frac{q\left(z_{\mathcal{H}_{\text{IB}}}\right)}{p\left(z_{\mathcal{H}_{\text{IB}}}\right)}\,\mathrm{d}z_{\mathcal{H}_{\text{IB}}}\,\mathrm{d}\mathcal{H} \\
&= \mathbb{E}_{p(\mathcal{H})}\left[\mathrm{KL}\left(p\left(z_{\mathcal{H}_{\text{IB}}}\mid\mathcal{H}\right)\,||\,q\left(z_{\mathcal{H}_{\text{IB}}}\right)\right)\right] \\
&\quad - \mathbb{E}_{p\left(z_{\mathcal{H}_{\text{IB}}}\mid\mathcal{H}\right)}\left[\mathrm{KL}\left(p\left(z_{\mathcal{H}_{\text{IB}}}\right)\,||\,q\left(z_{\mathcal{H}_{\text{IB}}}\right)\right)\right] \\
&\leq \mathbb{E}_{p(\mathcal{H})}\left[\mathrm{KL}\left(p\left(z_{\mathcal{H}_{\text{IB}}}\mid\mathcal{H}\right)\,||\,q\left(z_{\mathcal{H}_{\text{IB}}}\right)\right)\right],
\end{aligned}
\tag{1}
$$

where $q\left(z_{\mathcal{H}_{\text{IB}}}\right)$ is the variational approximation to $p\left(z_{\mathcal{H}_{\text{IB}}}\right)$. And the inequality is due to the fact that Kullback-Leibler divergence is non-negative. We assume that $q\left(z_{\mathcal{H}_{\text{IB}}}\right)$ is a noninformative distribution following VIB [20]. That is, we obtain $q\left(z_{\mathcal{H}_{\text{IB}}}\right)$ by aggregating the node representations in a fully perturbed graph. The noise $\epsilon_{\mathcal{H}} \sim \mathcal{N}\left(\mu_{\mathcal{H}},\sigma_{\mathcal{H}}^2\right)$ is sampled from the Gaussian distribution. $\mu_{\mathcal{H}},\sigma_{\mathcal{H}}^2$ are mean and variance of $\mathcal{H}_j$ in $\mathcal{H}$.

When we choose sum pooling as the readout function, we have:

$$
q\left(z_{\mathcal{H}_{\text{IB}}}\right) = \mathcal{N}\left(m_{\mathcal{H}}\mu_{\mathcal{H}}, m_{\mathcal{H}}\sigma_{\mathcal{H}}^2\right). \tag{2}
$$

This is because the summation of Gassian distributions is also a Gaussian distribution. Then, for $p\left(z_{\mathcal{H}_{\text{IB}}}\mid\mathcal{H}\right)$, we have:

$$
\begin{aligned}
&p\left(z_{\mathcal{H}_{\text{IB}}}\mid\mathcal{H}\right) \\
&= \mathcal{N}\left(m_{\mathcal{H}}\mu_{\mathcal{H}} + \sum_{j=1}^{m_{\mathcal{H}}}\lambda_j\mathcal{H}_j - \sum_{j=1}^{m_{\mathcal{H}}}\mu_{\mathcal{H}}\lambda_j, \sum_{j=1}^{m_{\mathcal{H}}}\left(1-\lambda_j\right)^2\sigma_{\mathcal{H}}^2\right).
\end{aligned}
\tag{3}
$$

Plug Equation 2 and Equation 3 into Equation 1 and we have:

$$
\begin{aligned}
&I\left(z_{\mathcal{H}_{\text{IB}}};\mathcal{H}\right) \\
&\leq \int p(\mathcal{H})\left(-\frac{1}{2}\log A_{\mathcal{H}} + \frac{1}{2m_{\mathcal{H}}}A_{\mathcal{H}} + \frac{1}{2m_{\mathcal{H}}}B_{\mathcal{H}}^2\right)\mathrm{d}\mathcal{H} \\
&+ \int \frac{1}{2}p(\mathcal{H})\log m_{\mathcal{H}}\,\mathrm{d}\mathcal{H} \\
&= \int p(\mathcal{H})\left(-\frac{1}{2}\log A_{\mathcal{H}} + \frac{1}{2m_{\mathcal{H}}}A_{\mathcal{H}} + \frac{1}{2m_{\mathcal{H}}}B_{\mathcal{H}}^2\right)\mathrm{d}\mathcal{H} + C,
\end{aligned}
\tag{4}
$$

where $A_{\mathcal{H}} = \sum_{j=1}^{m_{\mathcal{H}}}\left(1-\lambda_j\right)^2$ and $B_{\mathcal{H}} = \frac{\sum_{j=1}^{m_{\mathcal{H}}}\lambda_j(\mathcal{H}_j-\mu_{\mathcal{H}})}{\sigma_h}$. C is a constant and can be ignored in the optimization process.

C Proof of $\mathcal{L}_{pre}$

Proof. Regarding $I(Y;\mathcal{H}_{\text{IB1}},\mathcal{H}_{\text{IB2}})$, we consider $P_\theta\left(Y \mid \mathcal{H}_{\text{IB1}},\mathcal{H}_{\text{IB2}}\right)$ as the variational estimation of $P\left(Y \mid \mathcal{H}_{\text{IB1}},\mathcal{H}_{\text{IB2}}\right)$. Therefore, we can proceed with the following derivation:

$$\begin{aligned} I(Y;\mathcal{H}_{\text{IB1}},\mathcal{H}_{\text{IB2}}) &= \mathbb{E}_{(Y,\mathcal{H}_{\text{IB1}},\mathcal{H}_{\text{IB2}})} \log\left[\frac{P\left(Y \mid \mathcal{H}_{\text{IB1}},\mathcal{H}_{\text{IB2}}\right)}{P(Y)}\right] \\ &= \mathbb{E}_{(Y,\mathcal{H}_{\text{IB1}},\mathcal{H}_{\text{IB2}})} \log\left[\frac{P_\theta\left(Y \mid \mathcal{H}_{\text{IB1}},\mathcal{H}_{\text{IB2}}\right)}{P(Y)}\right] + \\ \mathbb{E}_{\mathcal{H}_{\text{IB1}},\mathcal{H}_{\text{IB2}}} & \log\left[KL\left(P\left(Y \mid \mathcal{H}_{\text{IB1}},\mathcal{H}_{\text{IB2}}\right) \| P_\theta\left(Y \mid \mathcal{H}_{\text{IB1}},\mathcal{H}_{\text{IB2}}\right)\right)\right]. \end{aligned} \tag{5}$$

Considering the non-negativity property of the Kullback-Leibler divergence, we can conclude that:

$$\begin{aligned} I(Y;\mathcal{H}_{\text{IB1}},\mathcal{H}_{\text{IB2}}) &\geq \mathbb{E}_{(Y,\mathcal{H}_{\text{IB1}},\mathcal{H}_{\text{IB2}})} \log\left[\frac{P_\theta\left(Y \mid \mathcal{H}_{\text{IB1}},\mathcal{H}_{\text{IB2}}\right)}{P(Y)}\right] \\ &= \mathbb{E}_{(Y,\mathcal{H}_{\text{IB1}},\mathcal{H}_{\text{IB2}})} \log\left[P_\theta\left(Y \mid \mathcal{H}_{\text{IB1}},\mathcal{H}_{\text{IB2}}\right)\right] + H(Y). \end{aligned} \tag{6}$$

As $H(Y)$ remains constant across all data, it can be omitted, resulting in the final formulation of this term:

$$\mathcal{L}_{pre} := \mathbb{E}_{(Y,\mathcal{H}_{\text{IB1}},\mathcal{H}_{\text{IB2}})} \log\left[P_\theta\left(Y \mid \mathcal{H}_{\text{IB1}},\mathcal{H}_{\text{IB2}}\right)\right]. \tag{7}$$

□

D Merged Molecular Explainability Injection

In this part, we elaborate the process of injecting explainability into the model. The input for the explanation module is the representation of the merged graph. Specifically, for a node, it receives the embedding of the node to output an importance score, and for an edge, it processes the concatenated embeddings of the two nodes connected by the edge to output the importance of the edge. Specifically, we get inspiration from the significant success of the post-hoc explanation methods of the graph neural networks [21–23] and design our explainers from three perspectives as follows.

- The first one is the **local mask-based perspective**. Specifically, local mask-based methods endeavour to multiply the features v'_{ai} and v'_{bi} with the corresponding masks (all are initialized to 1) p_{ai} and p_{bi} to get $v''_{ai} = v'_{ai}p_{ai}$ and $v''_{bi} = v'_{bi}p_{bi}$. Then, v''_{ai} and v''_{bi} is sent into the model to obtain the updated output ΔG_{solv}. Then they attempt to find the optimal score p_i by minimizing the difference between this processed output ΔG_{solv} and the label Y. To limit the size of explanatory subgraphs, they apply l_1 regularization to the value of mask. In this case, the loss function is:

$$\mathcal{L} = D\left(\Delta G_{\text{solv}}; Y\right) + \sum_i \lambda(p_{ai} + p_{bi}) \cdot \mathbf{1}^{\mathbf{T}}, \tag{8}$$

where D denotes the distance function; λ is the trade-off parameter. Followed by explanation methods such

as GSAT[1] [24] and PGExplainer [25].

- The second one is the **global mask-based perspective**, and its only difference from the first approach lies in the method of mask generation. Here, during the process of the adding the mask, the trainable mask p_{ai} and p_{bi} in local mask-based methods is replaced with a trainable MLP_ψ (*i.e.*, $p_{ai} = \text{MLP}_\psi(v'_{ai}), p_{bi} = \text{MLP}_\psi(v'_{bi})$). Meanwhile, following the theory of graph information bottleneck (GIB) [26, 27], these methods instantiates the *information constraint* (ℓ_p) proposed by [24], where ℓ_p is defined as:

$$\begin{aligned} \ell_{pa} &= \sum_i p_{ai} \log \frac{p_{ai}}{k} + (1 - p_{ai}) \log \frac{1 - p_{ai}}{1 - k} \\ \ell_{pb} &= \sum_i p_{bi} \log \frac{p_{bi}}{k} + (1 - p_{bi}) \log \frac{1 - p_{bi}}{1 - k}, \end{aligned} \tag{9}$$

where k is the pre-defined hyperparameter. After applying ℓ_p regularization to the value of mask, the expression of the final loss function is:

$$\mathcal{L} = D\left(\Delta G_{\text{solv}}; Y\right) + \alpha(\ell_{pa} + \ell_{pb}), \tag{10}$$

where D denotes the distance function; α is the trade-off parameter.

- The third one is the **gradient-based perspective**. For the v'_{ai} and v'_{bi}, these methods first calculate the absolute values of the features in the derivative of ΔG_{solv} *w.r.t* v'_{ai} and v'_{bi}. After that, their importance score p_{ai} and p_{bi} are defined as the normalized sum of these values. Then, at the end of each training phase, we mask some nodes and edges according to the above derivatives, and input the remain part to the next training phase. More formally:

$$p_{ai} = \left(|\frac{\partial \Delta G_{\text{solv}}}{\partial v'_{ai}}| \cdot \mathbf{1}^{\mathbf{T}} \right), p_{bi} = \left(|\frac{\partial \Delta G_{\text{solv}}}{\partial v'_{bi}}| \cdot \mathbf{1}^{\mathbf{T}} \right). \tag{11}$$

□

E Dataset analysis

As shown in the figure, we present the elemental composition and molecular weight distributions of solutes and solvents across five datasets. The most common elements are C, O, H, Cl, and F, while elements such as S, I, P, B, and Br appear with lower frequency. Carbon (C) is the most dominant element across all datasets. Notably, in the FreeSolv dataset, where all solvents are water, the elemental proportions differ slightly from the others. Furthermore, the molecular weight distributions exhibit clear differences among datasets. In the Abraham and CompSol-Exp datasets, most molecules have molecular weights below 100. In contrast, nearly half of the molecules in the MNSol dataset exceed 100, indicating a significant imbalance in molecular size distributions across datasets.

[1] The GSAT mentioned here refers to the GSAT in the post-explanation mode [24].

Table 1: Significance analysis of model performance across five datasets. We report the mean accuracy (%) and standard deviation in parentheses.

	**FreeSlove**	**CompSol**	**Abraham**	**CompSolv-Exp**	**MNSol**
**MMGNN**	$0.536_{(0.030)}$	$0.146_{(0.010)}$	$0.187_{(0.008)}$	$0.171_{(0.013)}$	$0.281_{(0.011)}$
**MMHNN**	$0.527_{(0.028)}$	$0.144_{(0.011)}$	$0.183_{(0.007)}$	$0.172_{(0.011)}$	$0.271_{(0.011)}$
**P-value**	1.04E-06	1.14E-02	1.53E-02	0.53E-01	1.41E-03

F Results significance analysis

Under the random split setting, where the dataset is randomly divided into training, validation, and test sets, we repeated the experiment 8 times and reported the mean, variance, and p-value of the accuracy (ACC), as shown in Table 1. Compared to the second-best model, MMHNN achieves a 2.10% improvement average on the test dataset expect CompSolv-Exp, with an average gain of approximately 0.05. All p-values are below 0.05, indicating that the performance improvement is statistically significant. Although the numerical margin may appear modest, the results are consistent and statistically robust.

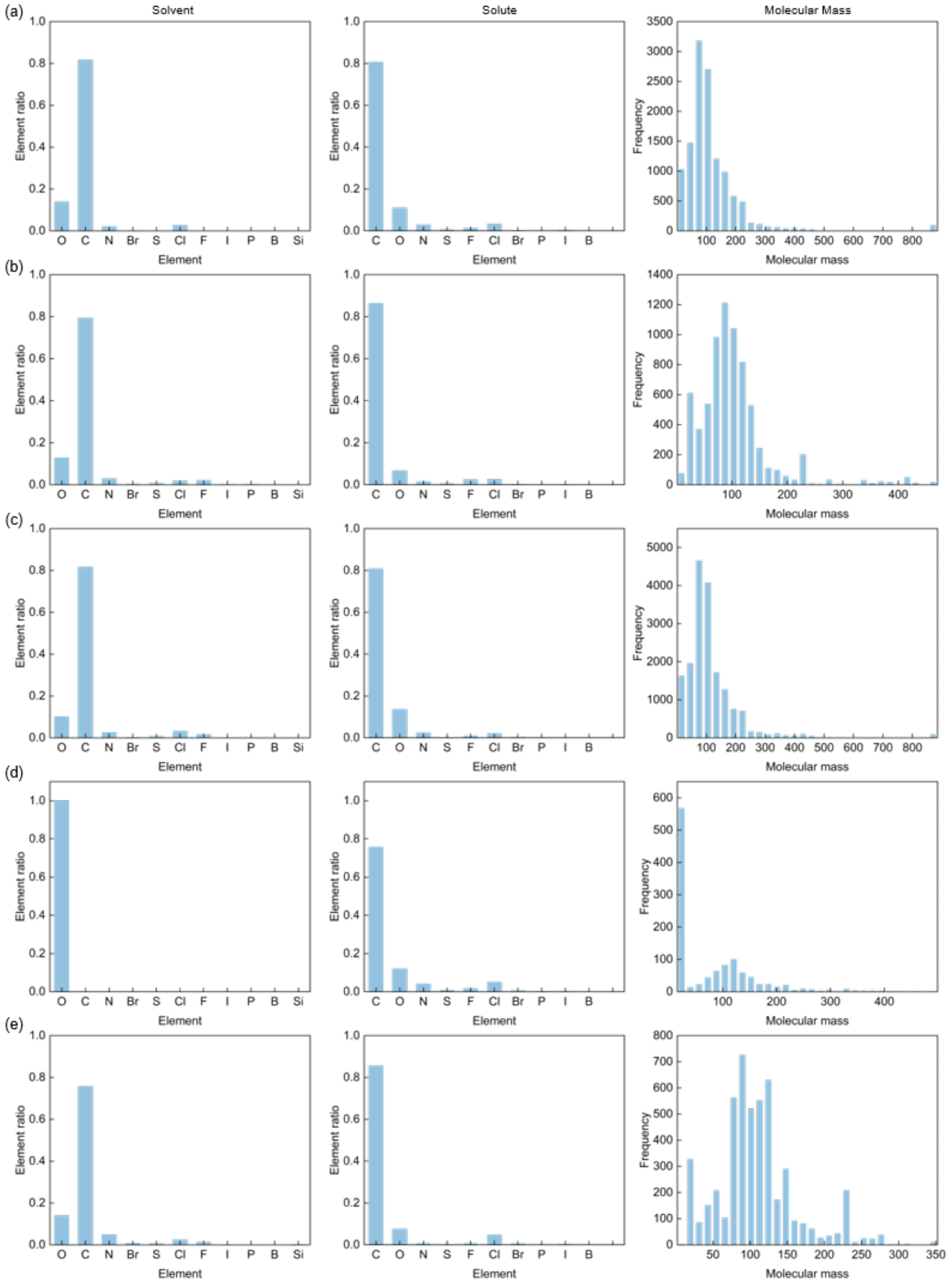

Figure 1: Comparison of elemental composition and molecular weight distributions of solutes and solvents across different datasets. (a) Abraham dataset; (b) CompSol dataset; (c) CompSol-Exp dataset; (d) Freesolve dataset; (e) MNSol dataset

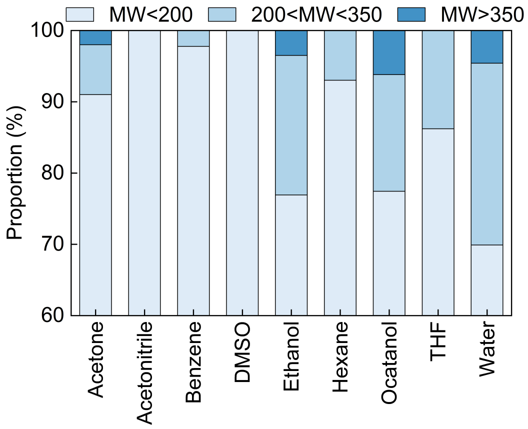

Figure 2: Comparative distribution of solute molecular weight proportions under different solvents.

References

1. Xia J, Lin H, Xu Y, et al. GNN Cleaner: Label Cleaner for Graph Structured Data. IEEE Transactions on Knowledge and Data Engineering 2023.
2. Wang B, Wang P, Zhang Y, et al. Towards Dynamic Spatial-Temporal Graph Learning: A Decoupled Perspective. In: *Proceedings of the AAAI Conference on Artificial Intelligence*. Vol. 38. 8. 2024:9089–97.
3. Wang B, Zhang Y, Shi J, et al. Knowledge expansion and consolidation for continual traffic prediction with expanding graphs. IEEE Transactions on Intelligent Transportation Systems 2023.
4. Fedik N, Zubatyuk R, Kulichenko M, et al. Extending machine learning beyond interatomic potentials for predicting molecular properties. Nat Rev Chem 2022;6:653–72.
5. Behler J. Constructing high-dimensional neural network potentials: a tutorial review. International Journal of Quantum Chemistry 2015;115:1032–50.
6. Behler J. Perspective: Machine learning potentials for atomistic simulations. The Journal of chemical physics 2016;145:170901.
7. Behler J and Parrinello M. Generalized neural-network representation of high-dimensional potential-energy surfaces. Physical review letters 2007;98:146401.
8. Yao K, Herr JE, Toth DW, McKintyre R, and Parkhill J. The TensorMol-0.1 model chemistry: a neural network augmented with long-range physics. Chemical science 2018;9:2261–9.
9. Glick ZL, Metcalf DP, Koutsoukas A, Spronk SA, Cheney DL, and Sherrill CD. AP-Net: An atomic-pairwise neural network for smooth and transferable interaction potentials. The Journal of Chemical Physics 2020;153:044112.
10. Pathak Y, Mehta S, and Priyakumar UD. Learning atomic interactions through solvation free energy prediction using graph neural networks. Journal of Chemical Information and Modeling 2021;61:689–98.
11. Lee N, Hyun D, Na GS, Kim S, Lee J, and Park C. Conditional Graph Information Bottleneck for Molecular Relational Learning. arXiv preprint arXiv:2305.01520 2023.
12. Metcalf DP, Koutsoukas A, Spronk SA, et al. Approaches for machine learning intermolecular interaction energies and application to energy components from symmetry adapted perturbation theory. The Journal of Chemical Physics 2020;152:074103.
13. Hildebrand JH. A HISTORY OF SOLUTION THEORY. Annual Review of Physical Chemistry 1981;32:1–23.
14. Klamt A and Eckert F. COSMO-RS: a novel and efficient method for the a priori prediction of thermophysical data of liquids. Fluid Phase Equilibria 2000;172:43–72.
15. Marenich AV, Olson RM, Kelly CP, Cramer CJ, and Truhlar DG. Self-consistent reaction field model for aqueous and nonaqueous solutions based on accurate polarized partial charges. Journal of Chemical Theory and Computation 2007;3:2011–33.

16. Vermeire FH and Green WH. Transfer learning for solvation free energies: From quantum chemistry to experiments. Chemical Engineering Journal 2021;418:129307.

17. Zhang DD, Xia S, and Zhang YK. Accurate Prediction of Aqueous Free Solvation Energies Using 3D Atomic Feature-Based Graph Neural Network with Transfer Learning. Journal of Chemical Information and Modeling 2022;62:1840–8.

18. Lim H and Jung Y. MLSolvA: solvation free energy prediction from pairwise atomistic interactions by machine learning. Journal of Cheminformatics 2021;13.

19. Low K, Coote ML, and Izgorodina EI. Explainable Solvation Free Energy Prediction Combining Graph Neural Networks with Chemical Intuition. J Chem Inf Model 2022;62:5457–70.

20. Alemi AA, Fischer I, Dillon JV, and Murphy K. Deep Variational Information Bottleneck. 2019. arXiv: 1612.00410 [cs.LG]. URL: https://arxiv.org/abs/1612.00410.

21. Ying Z, Bourgeois D, You J, Zitnik M, and Leskovec J. GNNExplainer: Generating Explanations for Graph Neural Networks. In: *NeurIPS*. 2019:9240–51.

22. Mou M, Pan Z, Zhou Z, et al. A transformer-based ensemble framework for the prediction of protein–protein interaction sites. Research 2023;6:0240.

23. Fang J, Li X, Sui Y, et al. EXGC: Bridging Efficiency and Explainability in Graph Condensation. CoRR 2024;abs/2402.05962.

24. Miao S, Liu M, and Li P. Interpretable and Generalizable Graph Learning via Stochastic Attention Mechanism. In: *International Conference on Machine Learning*. PMLR. 2022:15524–43.

25. Luo D, Cheng W, Xu D, et al. Parameterized Explainer for Graph Neural Network. In: *NeurIPS*. 2020.

26. Wu T, Ren H, Li P, and Leskovec J. Graph Information Bottleneck. In: *NeurIPS*. 2020.

27. Yu J, Xu T, Rong Y, Bian Y, Huang J, and He R. Graph Information Bottleneck for Subgraph Recognition. In: *ICLR*. OpenReview.net, 2021.
